# Paclitaxel and the dietary flavonoid fisetin: a synergistic combination that induces mitotic catastrophe and autophagic cell death in A549 non-small cell lung cancer cells

**DOI:** 10.1186/s12935-016-0288-3

**Published:** 2016-02-16

**Authors:** Anna Klimaszewska-Wisniewska, Marta Halas-Wisniewska, Tadeusz Tadrowski, Maciej Gagat, Dariusz Grzanka, Alina Grzanka

**Affiliations:** Department of Histology and Embryology, Faculty of Medicine, Nicolaus Copernicus University in Toruń, Collegium Medicum in Bydgoszcz, Karlowicza 24, 85-092 Bydgoszcz, Poland; Department and Clinic of Dermatology, Sexually Transmitted Diseases and Immunodermatology, Faculty of Medicine, Nicolaus Copernicus University in Toruń, Collegium Medicum in Bydgoszcz, M. Curie Skłodowskiej 9, 85-094 Bydgoszcz, Poland

**Keywords:** Fisetin, Paclitaxel, Combination therapy, Lung cancer, Mitotic catastrophe, Autophagy

## Abstract

**Background:**

The use of the dietary polyphenols as chemosensitizing agents to enhance the efficacy of conventional cytostatic drugs has recently gained the attention of scientists and clinicians as a plausible approach for overcoming the limitations of chemotherapy (e.g. drug resistance and cytotoxicity). The aim of this study was to investigate whether a naturally occurring diet-based flavonoid, fisetin, at physiologically attainable concentrations, could act synergistically with clinically achievable doses of paclitaxel to produce growth inhibitory and/or pro-death effects on A549 non-small cell lung cancer cells, and if it does, what mechanisms might be involved.

**Methods:**

The drug–drug interactions were analyzed based on the combination index method of Chou and Talalay and the data from MTT assays. To provide some insights into the mechanism underlying the synergistic action of fisetin and paclitaxel, selected morphological, biochemical and molecular parameters were examined, including the morphology of cell nuclei and mitotic spindles, the pattern of LC3-II immunostaining, the formation of autophagic vacuoles at the electron and fluorescence microscopic level, the disruption of cell membrane asymmetry/integrity, cell cycle progression and the expression level of LC3-II, Bax, Bcl-2 and caspase-3 mRNA.

**Results:**

Here, we reported the first experimental evidence for the existence of synergism between fisetin and paclitaxel in the in vitro model of non-small cell lung cancer. This synergism was, at least partially, ascribed to the induction of mitotic catastrophe. The switch from the cytoprotective autophagy to the autophagic cell death was also implicated in the mechanism of the synergistic action of fisetin and paclitaxel in the A549 cells. In addition, we revealed that the synergism between fisetin and paclitaxel was cell line-specific as well as that fisetin synergizes with arsenic trioxide, but not with mitoxantrone and methotrexate in the A549 cells.

**Conclusions:**

Our results provide rationale for further testing of fisetin in the combination with paclitaxel or arsenic trioxide to obtain detailed insights into the mechanism of their synergistic action as well as to evaluate their toxicity towards normal cells in an animal model in vivo. We conclude that this study is potentially interesting for the development of novel chemotherapeutic approach to non-small cell lung cancer.

**Electronic supplementary material:**

The online version of this article (doi:10.1186/s12935-016-0288-3) contains supplementary material, which is available to authorized users.

## Background

Lung cancer is one of the most commonly diagnosed cancer worldwide, where it also ranks first as a leading cause of cancer-related deaths among both men and women [1]. In the clinical practice, lung carcinomas are divided into two main histological types: small cell lung cancer (SCLC) and non-small cell lung cancer (NSCLC), where the other accounts for 75–85 % of total lung cancer cases [2]. Since a predominant number of NSCLC cases fail to be detected at the surgically resectable early stage of disease, therefore chemotherapy and radiotherapy remain the mainstay of the treatment for inoperable NSCLC patients. However, while there have been numerous drugs approved for use, they have often suffered from the limited clinical applicability due to the development of resistance by tumor cells and non-specific toxicity toward normal cells [3, 4]. One such a drug, paclitaxel (PTX), a taxane plant product derived from the bark of the pacific yew tree *Taxus brevifolia*, has demonstrated a clinically significant activity against a broad variety of tumor types and has become a first-line treatment for NSCLC [5]. Nevertheless, the administration of this drug at optimum doses and for a prolonged time has been hampered by the prevalence of serious side effects, such as neutropenia, neuropathy, and the acquisition of clinical resistance as well [6]. Recently, a considerable attention has been given to the identification of new therapeutic agents with synergistic effects with paclitaxel and other conventional cytostatic drugs as a promising direction to overcome the above mentioned drug drawbacks. In general, the combination therapies have proven to be more potent than monotherapy in the treatment of cancers. They not only potentiate the therapeutic efficacy of each agent alone and/or enable the use of reduced doses of a single drug but also decrease the possibility of the development of drug resistance [7, 8].

In this context, plant polyphenols, especially these from dietary sources, have recently received an increased scientific attention as the appropriate contenders to serve as a partner for traditional chemotherapeutic drugs. The dietary phytochemicals are commonly perceived as non-toxic, well-tolerated, easily available, inexpensive compounds that can target multiple cellular pathways [9, 10]. Indeed, there is evidence that these agents may potentiate the cytotoxic effects of chemotherapy and radiotherapy, protect normal cells from therapy-associated toxicity, increase a systemic bioavailability of cytostatic agents, and in some cases, even overcome chemoresistance [11, 12]. Despite the large number of the dietary polyphenols, not all of them share the same anticancer activity, hence a considerable attention of researchers has focused on the selected groups, including flavonoids. In several comparative studies, fisetin (3,3′,4′,7-tetrahydroxyflavone; FIS), a naturally occurring diet-based flavonoid, has stood alone as an effective anticancer agent against a broad spectrum of tumor cell lines, with some of the antitumor effects being achieved at physiologically relevant concentrations, and without affecting normal cells, including human bronchial epithelial (NHBE) cells [3, 13–18]. Therefore, the aim of this study was to investigate whether fisetin, at physiologically attainable concentrations, could act synergistically with clinically achievable doses of paclitaxel to produce growth inhibitory and/or pro-death effects on A549 non-small cell lung cancer cells, and if it does, what mechanisms might be involved. Here, we report the first experimental evidence on such synergistic action, which was, at least partially, ascribed to the induction of mitotic catastrophe and the autophagic cell death.

## Materials and methods

### Cell culture

The human non-small cell lung cancer cell line A549 and the human colon adenocarcinoma cell line LoVo were kindly provided by Dr. P. Kopinski (Department of Gene Therapy, Collegium Medicum in Bydgoszcz, Nicolaus Copernicus University, Poland) and Prof. P. Dziegiel (Department of Histology and Embryology, Wroclaw Medical University), respectively and both cell lines were cultured in Dulbecco’s Modified Eagle Medium (DMEM; Lonza; Verviers, Belgium). The human breast adenocarcinoma cell line MCF-7 was purchased from American Type Culture Collection (ATCC; HTB-22) and maintained in Minimum Essential Medium (MEM, Lonza; Verviers, Belgium) supplemented with 1 % non-essential amino acids (Sigma-Aldrich; St. Louis, MO, USA). The human non-small cell lung cancer cell line H1299 was bought from American Type Culture Collection (ATCC; CRL-5803) and these cells were grown in Roswell Park Memorial 1640 medium (RPMI 1640; Sigma-Aldrich; St. Louis, MO, USA). All media were supplemented with 10 % fetal bovine serum (PAA; Pasching, Austria) and 50 µg/ml gentamycin (Sigma-Aldrich; St. Louis, MO, USA) and all cultures were carried out in a humidified atmosphere of 95 % air and 5 % CO_2_ at 37 °C. After reaching 70–80 % confluence during the exponential growth, the cells were harvested with trypsin–EDTA solution (Sigma-Aldrich; St. Louis, MO, USA) and subcultured on 12- or 6-well plates (at the density of 0.11 × 10^6^ cells/well and 0.3 × 10^6^ cells/well, respectively) (BD Falcon; Franklin Lakes, NJ) for further experiments.

### Cell treatment

Stock solutions of fisetin and paclitaxel (Sigma-Aldrich; St. Louis, MO, USA) at concentration of 100 mM were prepared in 100 % dimethyl sulfoxide (Sigma-Aldrich; St. Louis, MO, USA), stored at 25 °C and serially diluted in complete growth medium immediately before use. After a 24-h incubation to allow cell attachment, paclitaxel (at concentrations of 0.1, 0.2, 0.3, 0.4, 0.5 µM for MTT assays and 0.1 µM for other experiments) or fisetin (at concentrations of 10, 20, 30, 40, 50 µM for MTT assays and 10 µM for other experiments) were added to cells for the indicated times as either single or combined agents at a fixed concentration ratio of 1:100. Preliminary screening was carried out to ascertain the incubation time and the treatment regimen—a sequential versus combined treatment (data not shown). The final concentrations of DMSO did not affect the cell viability (data not shown). Control cells were cultured under identical conditions, but without the addition of the tested agents.

### MTT assay

The cell viability was assessed using MTT colorimetric assay. The cells were seeded in 12-well plates in complete grown medium and 24 h later, the cells were treated with paclitaxel at doses from 0.1 to 0.5 μM and with fisetin at concentrations ranging from 10 to 50 μM, either alone or in a fixed ratio of 1:100, for the next 24 h. The MTT stock solution was made by dissolving 5 mg of thiazolyl blue tetrazolium bromide (MTT; Sigma-Aldrich; St. Louis, MO, USA) in 1 mL of phosphate-buffered saline (PBS) and sterilized by passage through a Whatman filter (Florham Park, NJ) with a pore size of 0.2 µm. After the drug treatment, the cells were once washed with PBS and incubated for 3 h (37 °C, 5 % CO_2_, 95 % air atmosphere) in a working solution of MTT, prepared by diluting a stock solution with DMEM without phenol red (Lonza; Verviers, Belgium) in the ratio 1:9. The surviving cells converted MTT to formazan that generated a blue-purple color when dissolved in acidic isopropanol. Dye absorbance was measured at 570 nm using spectrophotometer (Spectra Academy, K-MAC, Korea). The experiment was repeated six times and the cell viability was calculated as the percentage of MTT reduction, assuming the absorbance of control cells as 100 %. Under the used conditions, MTT assay allowed to estimate the loss in cell viability resulting from the inhibition of the cell proliferation, the increase in cell death or the sum of both processes.

### Drug interaction analysis

To evaluate whether the antitumor effects of the combination of fisetin and paclitaxel were synergistic, additive or antagonistic, the drug interactions were analyzed based on the combination index method of Chou and Talalay [19]. Using data obtained from MTT assays and CompuSyn software [20], the dose–effect curves for single agents and their combinations were generated and the combination index (CI) values for each dose and the corresponding effect level, referred to as the fraction affected (f_a_; the fraction of cells inhibited after the drug exposure, e.g. 0.5 when cell growth is inhibited by 50 %), were calculated. The resulting combination index offers a quantitative definition for an additive effect (CI = 1), synergism (CI < 1), and antagonism (CI > 1) in drug combinations [21]. Then, to provide a visual illustration of drug interactions, the F_a_–CI plot was constructed by simulating CI values over a range of f_a_ levels from 0.1 to 0.95.

### Annexin V/propidium iodide (PI) binding assay

To assess the extent of apoptosis, the Tali Apoptosis kit—Annexin V Alexa Fluor 488 and Propidium Iodide (Invitrogen/Life Technologies, Carlsbad, CA, USA) was used according to the manufacturer’s instructions. In short, after the treatment, the cells were collected from 6-well plates using trypsin–EDTA solution, centrifuged at 300×*g* for 8 min, resuspended in annexin binding buffer (ABB) and incubated with Annexin V Alexa Fluor 488 at room temperature (RT) in the dark for 20 min. Following the centrifugation at 300×*g* for 5 min, the cells were again resuspended in ABB and incubated with propidium iodide at RT in the dark for 5 min. The cells were analyzed using Tali image-based cytometer (Invitrogen/Life Technologies, Carlsbad, CA, USA). The data were quantified by FCS Express Research Edition software (version 4.03; De Novo Software, New Jersey, NJ, USA) and expressed as the percentage of cells in each population (viable Annexin V^−^/PI^−^; early apoptotic Annexin V^+^/PI^−^; late apoptotic Annexin V^+^/PI^+^; necrotic Annexin V^−^/PI^+^). The sum of the early and late apoptotic cells represented the total apoptosis.

### Cell cycle analysis

For DNA content analysis, the Tali Cell Cycle Kit (Invitrogen/Life Technologies, Carlsbad, CA, USA) was used according to the manufacturer’s instructions. Briefly, the treated cells were harvested from 6-well plates by trypsinization, rinsed with PBS, fixed in ice-cold 70 % ethanol at 4 °C, and left at −25 °C overnight. The next day, the cells were centrifuged at 1000×*g* for 5 min at 4 °C and washed with PBS. After the centrifugation at 500×*g* for 10 min at 4 °C, the cells were resuspended in the Tali Cell Cycle Solution containing propidium iodide (PI), RNase A, and Triton X-100. Following 30-min incubation at RT in the dark, the cells were analyzed using Tali image-based cytometer (Invitrogen/Life Technologies, Carlsbad, CA, USA) and the percentage of cells in each phase of the cell cycle was determined using FCS Express Research Edition software (version 4.03; De Novo Software, New Jersey, NJ, USA).

### Fluorescent staining of β-tubulin and cell nuclei

For spindle morphology assessment, the cells were seeded on glass cover slides in 12-well plates, permitted to adhere overnight and then treated with fisetin and/or paclitaxel. After the prefixation step with 1 mM bifunctional protein cross-linking reagent 3,30-dithiodipropionic acid (DTSP; Sigma-Aldrich, St. Louis, MO, USA) in Hank’s balanced salt solution (HBSS; Sigma-Aldrich, St. Louis, MO, USA) for 10 min, the cells were extracted in TsB (0.5 % Triton X-100; Serva, Heidelberg, Germany) in microtubule stabilizing buffer (MTSB; 1 mM EGTA, 4 % poly(ethylene glycol), 10 mM PIPES; Sigma-Aldrich, St. Louis, MO, USA) containing DTSP (dilution 1:50) for 10 min and rinsed with TsB for 5 min. Following the fixation of the cells with 4 % paraformaldehyde (Serva, Heidelberg, Germany) in MTSB for 15 min and three washing steps with PBS, the cells were incubated with 1 % bovine serum albumin (BSA; Sigma-Aldrich, St. Louis, MO, USA) diluted in tris-buffered saline (TBS; Sigma-Aldrich, St. Louis, MO, USA) for 15 min. β-tubulin was labeled using the mouse monoclonal antibody specific for β-tubulin (Sigma-Aldrich, St. Louis, MO, USA) diluted 1:65 in 1 % BSA-PBS (1 h, a moist chamber). This was followed by rinsing the cells three times in 1 % BSA-PBS and incubation with the goat anti-mouse IgG-TRITC secondary antibody (Sigma-Aldrich, St. Louis, MO, USA), diluted 1:85 in PBS (45 min, a moist chamber, in the dark). To assess the nuclear morphology, the slides were incubated with 4′,6-diamino-2-phenylindole (DAPI, diluted 1:20,000 in distilled water; Sigma-Aldrich; St. Louis, MO) for 10 min in the dark. Finally, the slides were rinsed three times with distilled water, mounted with Aqua-Poly/Mount (Polysciences; Warrington, PA) and analyzed using Nikon Eclipse E800 fluorescence microscope and NIS-Elements 4.0 software (all from Nikon; Tokyo, Japan). At least 100 mitotic cells from three independent experiments were counted to determine the percentage of cells with the bipolar, multipolar or monopolar spindles.

### Transmission electron microscopy

To detect the presence of autophagic vacuoles in transmission electron microscope (TEM), the treated cells were harvested from 6-well plates, fixed with 3.6 % (v/v) glutaraldehyde (Polysciences; Warrington, PA) in 0.1 M sodium cacodylate buffer (pH 7.4; Roth; Karlsruhe, Germany) for 30 min at RT and washed three times in 0.1 M sodium cacodylate buffer. Then, the cells were entrapped within fibrin clots, which were formed by the vigorous mixing of cell pellets with the equal volumes of the fibrinogen (3 mg/ml; Sigma-Aldrich; St. Louis, MO, USA) and thrombin solution (50 U/ml; Biomed-Lublin; Lublin, Poland). Afterwards, the samples were post-fixed with 1 % (w/v) osmium tetroxide (Serva; Heidelberg, Germany) in 0.1 M sodium cacodylate buffer for 1 h at RT, rinsed three times with 0.1 M sodium cacodylate buffer, dehydrated through a graded ethanol (30–90 %; POCH S.A., Gliwice, Poland) and acetone (90–100 %; POCH S.A., Gliwice, Poland) series, infiltrated with increasing ratios of epoxy resin (Epon 812; Roth; Karlsruhe, Germany) with hardeners (DBA and MNA; Roth; Karlsruhe, Germany): 100 % acetone and finally embedded in gelatin capsules (Ted Pella, Inc.; Redding, CA) filled with pure epoxy resin with DMP-30 (Roth; Karlsruhe, Germany). The polymerization of the resin occurred at 37 °C for 24 h, and then at 65 °C for 120 h. The selected parts of the material were cut into ultra-thin sections using Reichert Om U3 ultramicrotome, placed on copper grids (Sigma-Aldrich; St. Louis, MO, USA), stained with 1 % uranyl acetate (Ted Pella, Inc.; Redding, CA) and examined with JEM-100CX transmission electron microscope (JEOL; Tokyo, Japan).

### Detection and quantification of acidic vesicular organelles with acridine orange staining

To detect the development of acidic vesicular organelles (AVOs), which are the hallmark of autophagy, the vital staining of A549 cells with acridine orange (AO; Sigma-Aldrich; St. Louis, MO, USA) was performed. The cells were seeded on coverslips in 12-well plates and after the attachment, they were incubated with fisetin and/or paclitaxel. Then, the cells were stained with medium containing 1 μg/ml AO for 15 min at 37 °C, washed twice in PBS and immediately examined with Nikon Eclipse E800 fluorescence microscope and NIS-Elements 4.0 software (all from Nikon; Tokyo, Japan). The cytoplasm and nucleus of AO-stained cells fluoresced bright green, whereas the acidic autophagic vacuoles fluoresced bright red, as described previously [22]. To quantify the development of AVOs, the cell pellets were stained with AO (1 μg/mL) for 15 min at 37 °C, washed twice with PBS and instantly analyzed with Tali Image-Based Cytometer (Invitrogen/Life Technologies, Carlsbad, CA, USA) and FCS Express Research Edition software (version 4.03; De Novo Software, New Jersey, NJ, USA). Approximately equal amounts of cells were measured for each sample and the geometric mean of red fluorescence intensity was used to quantify the responses. To inhibit autophagy, the cells were pretreated with 100 nM bafilomycin A1 (Baf A1) for 4 h, followed by washing with PBS and subsequent incubation in the absence or presence of the tested compounds for the indicated period of time.

### Immunofluorescent staining of LC3-II

To examine the intensity and the pattern of LC3-II immunostaining, the cells were seeded on glass cover slides in 12-well plates and left to attach overnight. After the treatment with fisetin and/or paclitaxel, the cells were fixed with 4 % paraformaldehyde (Serva; Heidelberg, Germany) in PBS for 30 min, washed three times with PBS, permeabilized with 0.25 % Triton X-100 (Serva; Heidelberg, Germany) in PBS for 5 min and blocked with 1 % BSA (Sigma-Aldrich; St. Louis, MO, USA) in PBS (BSA–PBS pH 7.6) for 20 min. The staining of LC3-II was performed using the rabbit anti-LC3-II antibody (Thermo Scientific; Rockford, USA) diluted 1:2000 in 1 % BSA–PBS (1 h, RT, a moist chamber). After rinsing three times with 1 % BSA–PBS, the cells were incubated with Alexa Fluor 488-labeled goat anti-rabbit secondary antibody (Life Technologies Corp.; Carlsbad, CA, USA) diluted 1:100 in PBS (60 min, RT, a moist chamber in the dark). Following three washing steps with PBS, the cell nuclei were counterstained with DAPI (diluted 1:20,000 in distilled water; Sigma-Aldrich; St. Louis, MO, USA) for 10 min. Finally, the slides were rinsed three times with distilled water, mounted with Aqua-Poly/Mount (Polysciences; Warrington, PA) and examined using Nikon Eclipse E800 fluorescence microscope and NIS-Elements 4.0 software (all from Nikon; Tokyo, Japan).

### Quantitative real-time PCR analysis

To determine the expression level of LC3-II, Bax, Bcl-2 and caspase-3, SYBR green-based quantitative real-time PCR was performed using LightCycler 2.0 Instrument (Roche Applied Science; Mannheim, Germany) and LightCycler Software Version 4.0. Total RNA from the A549 cells was prepared by using the Total RNA kit (A&A biotechnology; Gdynia, Poland) according to the manufacturer’s protocol. The reverse transcription and quantitative PCR reactions were performed in a single 20-μl LightCycler capillary (Roche Applied Science; Mannheim, Germany) as a one-step real-time qRT-PCR with TranScriba reverse transcriptase and Master Mix SYBR (TranScriba-qPCR Master Mix SYBR kit; A&A biotechnology; Gdynia, Poland) as specified by the manufacturer. The total reaction mixture (20 µl) contained 65 ng of RNA and 0.2 μM of each primer in addition to the TranScriba-qPCR Master Mix SYBR kit components. The sequences of primers were as follows: LC3-II forward 5′-CGGTGATAATAGAACGATACAAGG-3′; LC3-II reverse 5′-CTGAGATTGGTGTGGAGACG-3′; Bax forward 5′-AGATGTGGTCTATAATGCGTTTTCC-3′, Bax reverse 5′-CAGAAGGCACTAATCAAGTCAAGGT-3′; Bcl-2 forward 5′-AACATCGCCCTGTGGATGAC-3′, Bcl-2 reverse 5′-AGAGTCTTCAGAGACAGCCAGGAG-3′; caspase-3 forward 5′-TGGTTCATCCAGTCGCTTTG-3′, caspase 3 reverse 5′-CATTCTGTTGCCACCTTTCG-3′. One cycle of reverse transcription was carried out for 10 min at 50 °C, one cycle of denaturation for 3 min at 95 °C, and 40 cycles of denaturation for 15 s at 95 °C, followed by annealing and elongation for 30 s at 57–60 °C (depending on the primer’s melting temperature). Relative mRNA expression levels of LC3-II, caspase-3, Bax, and Bcl-2 were quantified using the ΔΔCt method (2^−ΔΔCt^ method) [23] and the results were normalized to the expression of the housekeeping gene glucose 6-phosphate dehydrogenase (G6PD) or glyceraldehyde-3-phosphate dehydrogenase (GAPDH) and presented as a fold difference relative to a calibrator sample (untreated A549 cells).

### Statistical analysis

Statistical analysis was performed with GraphPad Prism software (GraphPad Software, San Diego, CA). The nonparametric Mann–Whitney U test was used to compare the differences between experimental points, and the significance level was set to p < 0.05. Data are presented as mean ± standard deviation (SD).

## Results

### Fisetin acts synergistically with paclitaxel to decrease the viability of A549 cells

**Fig. 1:**
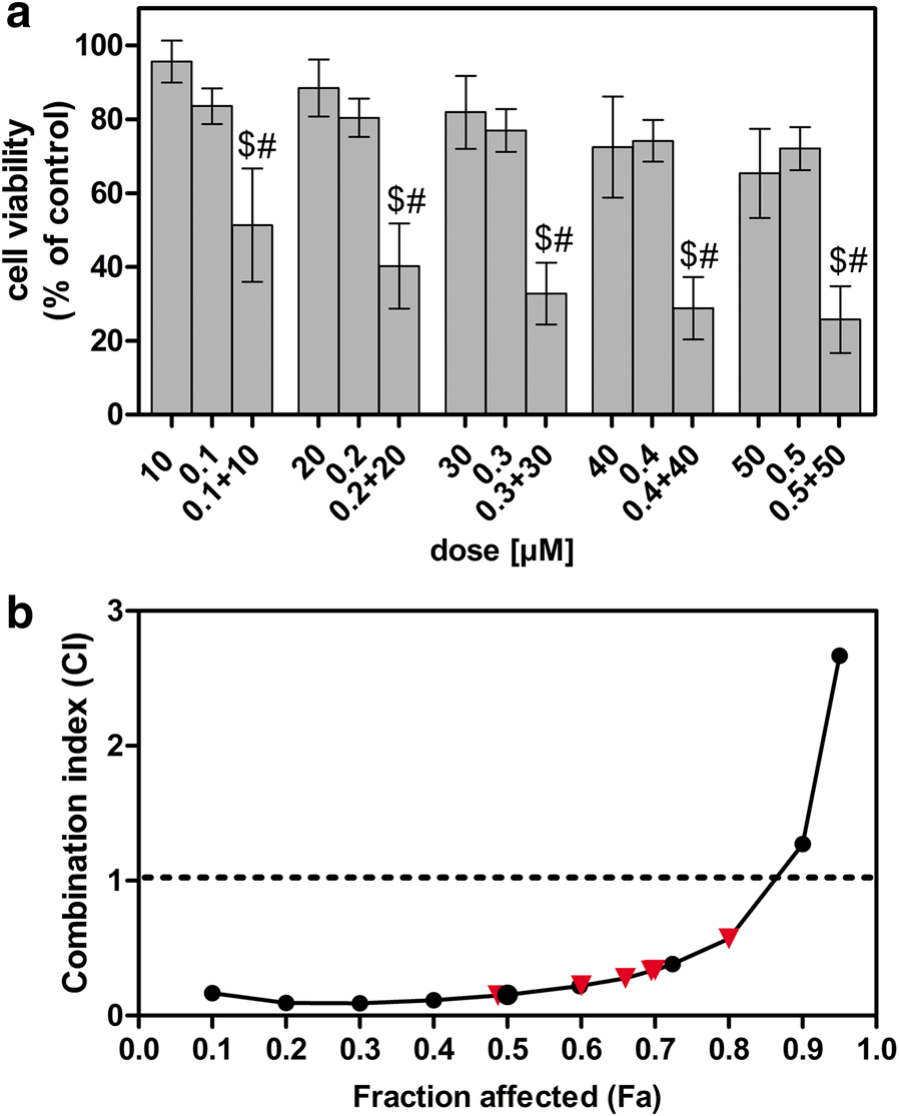
The combined effect of fisetin (FIS) and paclitaxel (PTX) on the A549 cells. **a** The cells were treated with various concentrations of PTX (0.1, 0.2, 0.3, 0.4, 0.5 μM) and FIS (10, 20, 30, 40, 50 µM), either alone or in a fixed ratio of 1:100, for 24 h. Cell viability was determined by MTT colorimetric assay. Data are expressed as a percentage of the control. Symbols ^$^ and ^#^ indicate statistically significant differences compared with FIS or PTX treatment alone, respectively (p < 0.05; Mann–Whitney U test). All values represent the mean ± standard deviation of six independent experiments. **b** Combination index plot (F_a_–CI plot) for FIS and PTX co-treatment in A549 cells. CI values are plotted as a function of the fractional inhibition (f_a_) of cell viability/growth by computer simulation (CompuSyn software) from 0.1 to 0.95. Triangles represent CI values derived from the actual experimental points. CI < 1 designates synergism, CI = 1 indicates additivity (denoted by a *dashed line*), and CI > 1 represents antagonism

In the first instance, we performed MTT assays to determine the cytotoxicity of fisetin, paclitaxel and their combinations on the human non-small cell lung cancer A549 cells and to evaluate whether FIS and PTX present additive, synergistic, or antagonistic effects on these cells. For this purpose, we chose the concentrations of fisetin (10, 20, 30, 40, 50 μM), which are into or close to the range of attainable serum levels of FIS [3, 24] and the doses of paclitaxel (0.1, 0.2, 0.3, 0.4, 0.5 μM), which are considered as clinically achievable [25, 26]. Consistent with the previous reports, PTX or FIS exhibited a dose-dependent growth inhibitory effect on the A549 cells (Fig. 1a). It should be pointed that the low concentrations of fisetin (≤20 μM,) had a little, if any, impact on the cell viability, allowing 95.63 % ± 5.073 (10 μM) and 88.46 % ± 7.73 (20 μM) of the A549 cells to survive (Fig. 1a). Furthermore, PTX at concentrations up to 0.5 μM failed to substantially decrease the cancer cell viability, enabling 83.55 % ± 4.84 to 72.05 % ± 5.81 of cells to remain alive (Fig. 1a), the results of which are in agreement with the earlier reports from the A549 cell line [27–30]. However, the combination treatments resulted in a significantly greater loss of the cell viability compared to the individual agents, suggesting the synergistic growth inhibitory effect of FIS and PTX on the A549 cells (Fig. 1a). To confirm this possibility, the interactions between FIS and PTX were analyzed with the median-effect principle of Chou and Talalay [19]. The mass-action law parameters, including *m*, D_*m*_, and *r* values that represent the slope of the median-effect plot (shape parameter), the median-effect dose (potency parameter, such as IC_50_, half maximal inhibitory concentration), and linear correlation coefficient of the median-effect plot (conformity parameter), respectively, [21] are presented in Table 1. The CI values at actual experimental points and different effect levels form 0.1 (IC_10_) to 0.90 (IC_90_) were calculated and shown in Tables 2 and 3, respectively. A graphical representation of the obtained results was combination index curve (F_a_–CI plot), in which the CI values between 0 and 2.7 were plotted against the corresponding effect levels (Fig. 1b). For the actual experimental points, the CI values ranged from 0.15 to 0.38 at effect levels from 0.50 (IC_50_) to 0.72 (IC_72_), indicating the strong synergistic (CI < 0.3) and synergistic (0.3 ≤ CI < 0.7) interactions between FIS and PTX in the doses chosen for this study (Table 2). Furthermore, the analysis of the CIs for a wide range of effect levels revealed that the interaction of FIS and PTX was strongly synergistic at f_a_ values ranging from 0.1 (IC_10_) to 0.65 (IC_65_) and became synergistic and moderately synergistic (0.7 ≤ CI < 0.85) at f_a_ levels between 0.7 (IC_70_) and 0.85 (IC_85_), approaching antagonism at f_a_ values above 0.95 (IC_95_) (Fig. 1b). Importantly, the maximal synergism was found when 10 μM FIS was combined with 0.1 μM PTX (Table 2), and since these concentrations represent both physiologically achievable level of the flavonoid and clinically relevant dose of the drug, therefore we selected them for further drug combination studies on mechanistic aspects, and unless otherwise stated, the incubation time was 24 h.

**Table 1:** The mass-action law parameters for fisetin and/or paclitaxel in A549 cells

Compound	M	D_m_	r	n
FIS	1.69	74.88	0.99	5
PTX	0.36	9.63	0.99	5
FIS + PTX	0.63	10.87	0.99	5

The data obtained from MTT assays were subjected to automated calculation of *m*, D*m* (µM) and *r* parameters using CompuSyn software. The parameters *m*, D_*m*_, and *r* are the slope, the median-effect dose, and the linear correlation coefficient of the median-effect plot, which signify the shape of the dose–effect curve, the potency (IC_50_), and the conformity of the data to the mass-action law, respectively; *n* is the number of sets of dose–effect relationship experiments that were conducted

*FIS* fisetin, *PTX* paclitaxel

**Table 2:** The combination index values at actual experimental points for the combination of fisetin and paclitaxel

Drug combination		CI	F_a_	Interaction type
FIS (µM)	PTX (µM)			
10	0.1	0.15	0.50	Strong synergism
20	0.2	0.22	0.60	Strong synergism
30	0.3	0.28	0.66	Strong synergism
40	0.4	0.33	0.70	Synergism
50	0.5	0.38	0.72	Synergism

The CI values were calculated according to the method of Chou–Talalay using CompuSyn software. CI < 1 represents synergism, with CI of 0.1–0.3 indicating strong synergism, 0.3–0.7 indicating synergism, CI of 0.7–0.85 indicating moderate synergism, CI of 0.85–0.90 indicating slight synergism, CI of 0.90–1.10 indicating a nearly additive effect, CI of 1.10–1.20 indicating a slight antagonism, and CI of 1.20–1.45 indicating a moderate antagonism

*Fa* fraction affected by dose, *FIS* fisetin, *PTX* paclitaxel

**Table 3:** The combination index values at different effect levels for the combination of fisetin and paclitaxel

F_a_	0.1	0.2	0.3	0.4	0.5	0.6	0.7	0.8	0.9
CI	0.17	0.09	0.09	0.12	0.16	0.22	0.34	0.57	1.27

The CI values were calculated according to the method of Chou–Talalay using CompuSyn software. CI < 1 represents synergism, with a CI of 0.1–0.3 indicating strong synergism, 0.3–0.7 indicating synergism, and CI of 0.7–0.85 indicating moderate synergism

**Fig. 2:**
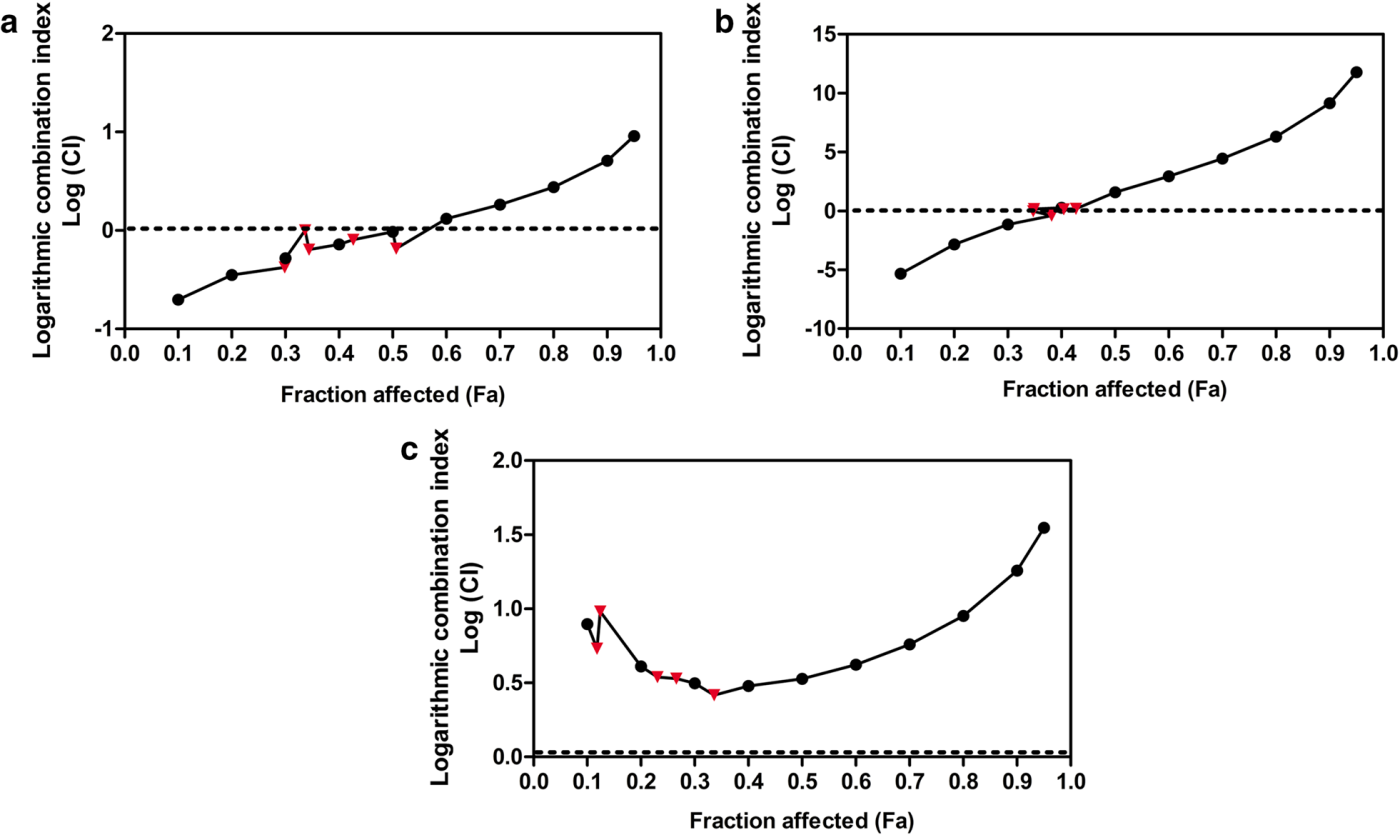
The combined effect of fisetin (FIS) and paclitaxel (PTX) on other solid tumor cell lines. Logarithmic combination index plot (F_a_-log(CI) plot) for FIS and PTX co-treatment in **a** the human colon adenocarcinoma LoVo cells **b** the human non-small cell lung cancer H1229 cells **c** the human breast adenocarcinoma MCF-7 cells. CI values (logarithmic) are plotted as a function of the fractional inhibition (f_a_) of cell viability/growth by computer simulation (CompuSyn software) from 0.1 to 0.95. Triangles represent CI values derived from the actual experimental points. In the F_a_-log(CI) plot, the synergism is indicated by a negative value (log(CI) < 0), antagonism is indicated by a positive value (log(CI) > 0), and additive effect (denoted by a dashed line) is indicated by 0 (log(CI) = 0)

In addition, it seemed relevant to determine if the synergistic interaction of FIS and PTX was specific to lung adenocarcinoma A549 cells. Thus, the effect of FIS and PTX applied separately or in combination was also assessed in other human solid tumor cell lines, including H1299 (large cell lung carcinoma), LoVo (colon adenocarcinoma) and MCF-7 (breast adenocarcinoma). As shown in Fig. 2, the combination treatments produced the various degrees of synergism, an additive effect or antagonism in a cell type-specific manner. In the case of the LoVo cells, the F_a_–CI plot showed synergism at f_a_ levels ranging from 0.1 to 0.4, the moderate synergism and the nearly additive effect for f_a_ values amounted to 0.45 and 0.5, respectively, and antagonism above these values (Fig. 2a). The CIs for the all actual experimental points indicated synergism or the nearly additive effects (Fig. 2a, triangles). A similar but less promising interaction pattern was found for the combination of FIS and PTX in the H1299 cells (Fig. 2b). There was a tendency to turn toward the additive effect and subsequently toward the increasing antagonism at lower values of f_a_ than those observed in the case of the LoVo cell line. The CI values for 2 actual experimental points fell within the ambit of synergism and the nearly additive effect and for others indicated antagonism (Fig. 2b, triangles). In turn, the F_a_–CI plot drawn up for the MCF-7 cells showed the strong antagonism in the whole range of effect levels (Fig. 2c).

It is also noteworthy that in our preliminary experiments, we also tested the combinations of fisetin with other drugs with a potential or proven activity against NSCLC. The evaluation of the cytotoxicity of fisetin in the combination with, inter alia, mitoxantrone (MIT), methotrexate (MTX), and arsenic trioxide (ATO) against A549 cells was performed (Additional file 1: Figure S1).

Collectively, our results showed that FIS synergized with PTX or ATO, but not MIT or MTX in A549 cells as well as that the synerisctic effect of FIS and PTX was cell line-specific.

### The combination of FIS and PTX induced apoptosis at relatively low level but a massive appearance of the enlarged mononucleated and multinucleated cells

**Fig. 3:**
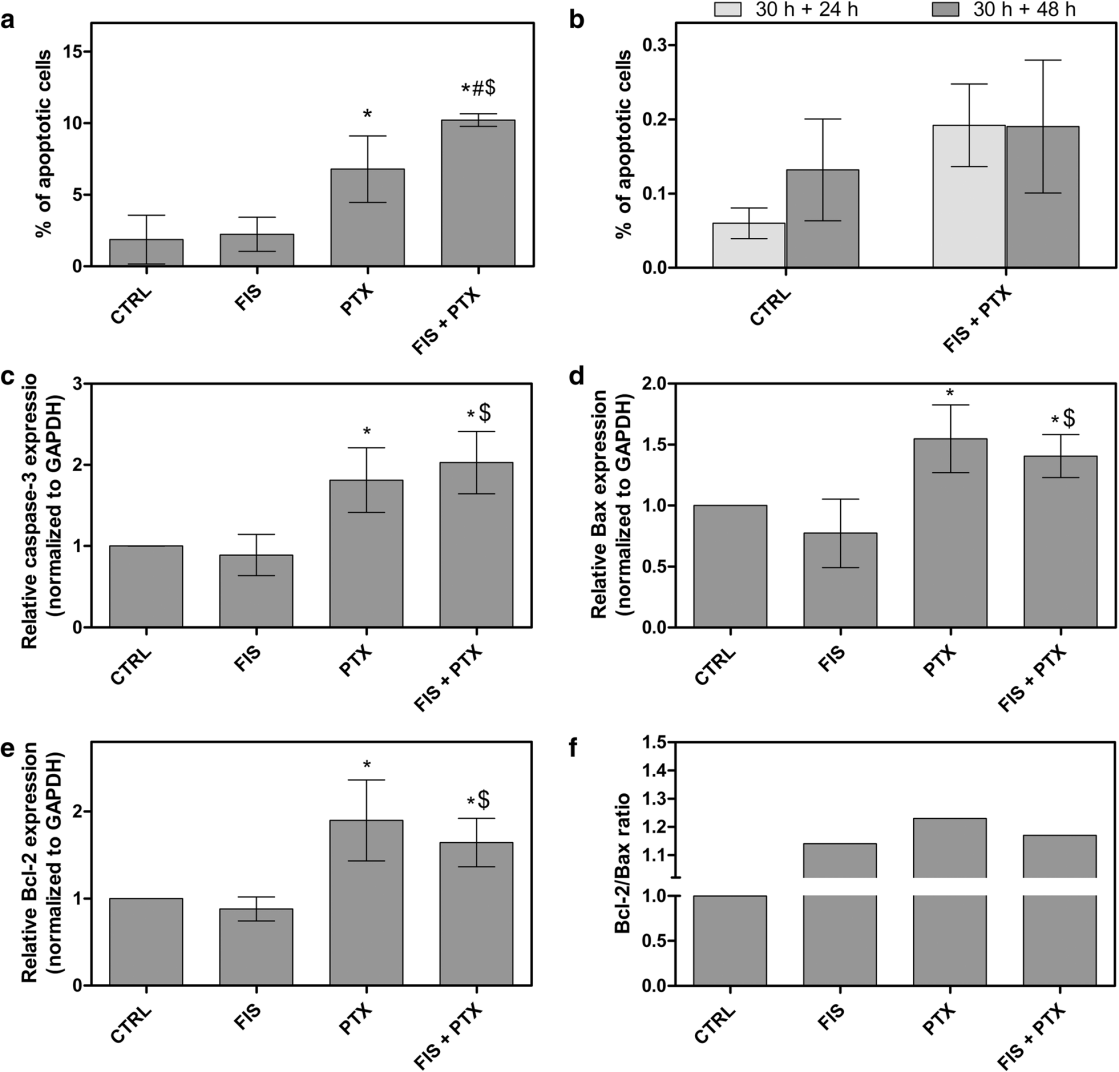
The effect of fisetin (FIS) and/or paclitaxel (PTX) on apoptosis of A549 cells. **a**, **b** Annexin V/PI assay by image-based cytometry. The sum of the early and late apoptotic cells represented the total apoptosis. **a**, **c**, **d**, **e** The A549 cells were treated with 10 µM FIS and/or 0.1 µM PTX for 24 h. **b** In another set of experiment, the cells were treated with the combination of 10 µM fisetin and 0.1 µM paclitaxel for 30 h, followed by post-treatment incubation in a drug-free medium for the next 24 or 48 h. Real-time PCR measurement of **c** caspase-3, **d** Bax, **e** Bcl-2 mRNA expression in A549 cells. The expression was normalized to GAPDH and presented as a fold difference relative to a calibrator sample (untreated A549 cells; designated as 1). **f** Bcl-2/Bax ratio. Control cells (CTRL) were cultured under identical conditions, but without the addition of the tested agents. Asterisks represent statistically significant differences from control cells (p < 0.05; Mann–Whitney U test). Symbols ^$^ and ^#^ indicate statistically significant differences compared with FIS or PTX treatment alone, respectively (p < 0.05; Mann–Whitney U test). The data are representative of five (**a**, **b**) or three (**c**–**e**) independent experiments and presented as mean and standard deviation

**Fig. 4:**
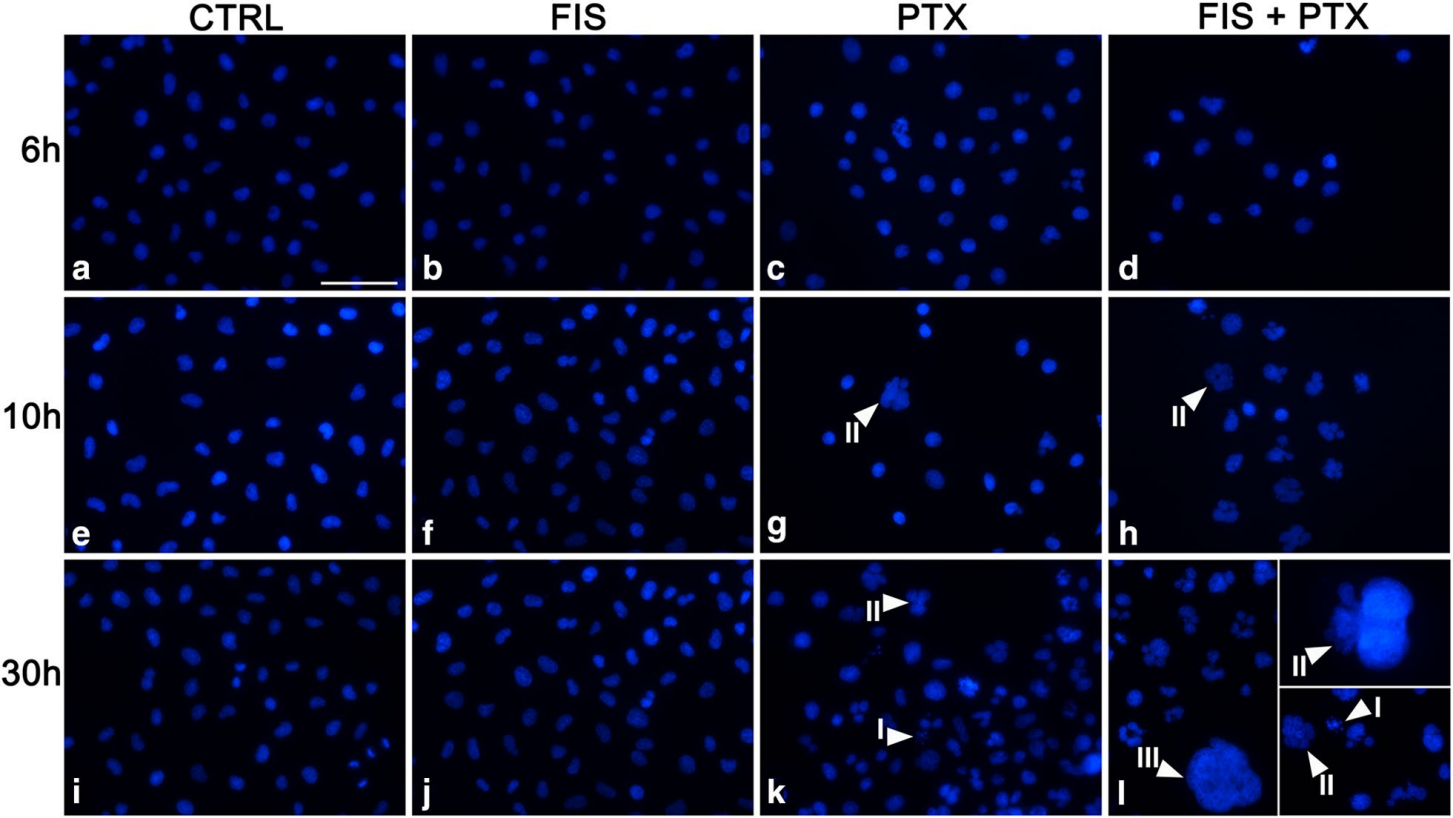
The assessment of the nuclear morphology using DAPI staining and fluorescence microscopy. The A549 cells were incubated with 10 µM fisetin (FIS), 0.1 µM paclitaxel (PTX) either as single agents or in the combination for 6, 10 or 30 h. Micrographs are representative of three independent experiments. *Arrowheads* indicate: (*I*) the shrunken or fragmented nuclei with chromatin condensation; the enlarged multinucleated (*II*) or mononucleated (*III*) cells. *Bar* 50 µm

To elucidate the mechanism underlying a synergistic cytotoxicity between FIS and PTX in the A549 cells, the apoptotic cell death was first measured by Annexin V/PI assay. As shown in Fig. 3a, the combined treatment yielded a higher level of Annexin V-positive cells than either agent alone, however even then the percentage of these cells was relatively low and did not exceed 11 %. Despite such a 1.5-fold increase of the number of the apoptotic cells, when compared to PTX alone, their number did not reflect the cytotoxicity of the combination of the agents which MTT assay indicated on. Moreover, the expression level of caspase-3 mRNA increased only twofold (2.028 ± 0.38) after the combined treatment, thus it correlated with a low fraction of the apoptotic cells (Fig. 3c). In addition, there were no statistically significant differences between PTX-treated and co-treated cells (Fig. 3c). Furthermore, both Bax and Bcl-2 mRNAs were upregulated by PTX with or without FIS, but the expression of Bcl-2 remained higher than that of Bax (Fig. 3d, e). Therefore, the Bcl-2/Bax ratios were greater than 1 in the cells exposed to PTX, FIS or FIS/PTX and amounted to 1.23, 1.14 and 1.17, respectively (Fig. 3f). The ratio of Bcl-2 to Bax is considered to be more important than expression levels of individual proteins in determining the susceptibility of cells to apoptosis. The Bcl-2/Bax ratio less than 1 makes cells susceptible to apoptosis, and when this ratio is higher than 1, the antiapoptotic signal should prevail among the cells [31, 32]. Similar as in the case of caspase-3, there was a lack of significant differences in the expression level of Bax and Bcl-2 between PTX-treated and co-treated cells (Fig. 3d, e). The quantitative analyses were further supplemented by the qualitative assessment of the nuclear morphology using fluorescence microscope and DAPI staining (Fig. 4**)**. We looked for characteristic features of apoptosis, such as the nuclear shrinkage and fragmentation as well as the chromatin condensation in the A549 cells treated with FIS and/or PTX and we found them in both PTX-treated and co-treated populations, nevertheless the apoptotic cells did not prevail in any group, being only occasionally seen (Fig. 4k, l—I). Furthermore, no detectable level of apoptosis was seen by image-based cytometer when FIS and PTX were administered to the A549 cells simultaneously for a period of 48 or 72 h (data not shown). Instead, there was a marked increase in the average cell size compared to control cells, from 12 ± 1 µm to 14.67 ± 0.58 µm (at 48 h) and from 13.33 ± 1.53 µm to 16 ± 1 µm (at 72 h) (data not shown). All these data allow us to assume that the alternative cell death mechanism other than apoptosis was involved in the synergistic action of FIS and PTX. Indeed, we observed a pronounced increase of the number of the enlarged mononucleated and multinucleated cells in the cell populations incubated with paclitaxel alone and we ascertained that such a drug effect was potentiated by fisetin in a time-dependent fashion (Fig. 4g, h, k, l—II, III). These results prompt us to perform the cell cycle analysis, since the observed morphological changes were reminiscent of those previously described for the mitotic cell death (mitotic catastrophe; MC).

### The A549 cells underwent a transient G2/M arrest followed by polyploidy and aneuploidy, but not apoptosis after the simultaneous treatment with FIS and PTX

**Fig. 5:**
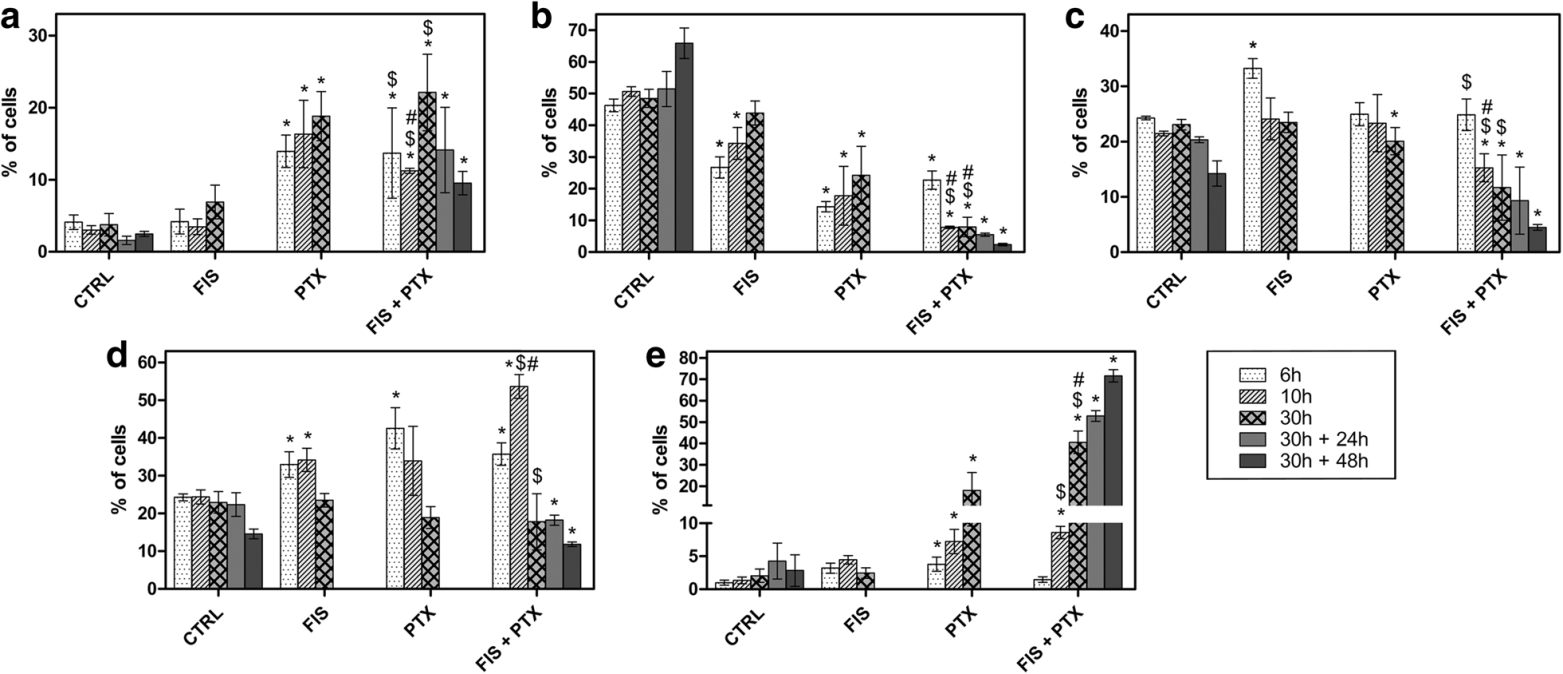
The cytometric analysis of cell cycle distribution using propidium iodide staining. The percentage of cells **a** with <4N DNA content, **b** in G0/G1 phase, **c** in S phase, **d** in G2/M phase, and **e** with >4N DNA content. The A549 cells were treated with 10 µM fisetin (FIS) and/or 0.1 µM paclitaxel (PTX) for 6, 10 or 30 h. In another set of experiment, the cells were treated with the combination of 10 µM fisetin and 0.1 µM paclitaxel for 30 h, followed by post-treatment incubation in a drug-free medium for the next 24 or 48 h. Control cells (CTRL) were cultured under identical conditions, but without the addition of the tested agents. Asterisks represent statistically significant differences from control cells (p < 0.05; Mann–Whitney U test). Symbols ^$^ and ^#^ indicate statistically significant differences compared with FIS or PTX treatment alone, respectively (p < 0.05; Mann–Whitney U test). The data are representative of five independent experiments and presented as mean and standard deviation

In order to determine the cell cycle effects of FIS and/or PTX, DNA content was measured using PI staining and image-based cytometer at three time points: at the completion of the 6, 10 and 30-h incubation with the tested compounds. In the first time point, there was a significant increase in the mean percentage of cells with DNA content corresponding to S phase of the cell cycle following the single treatment with fisetin (Fig. 5c). Furthermore, the combination of paclitaxel and fisetin resulted in the accumulation of cells from G0/G1 phase into G2/M, but the increase in G2/M arrest was approximately equivalent to that produced by PTX and FIS alone (Fig. 5b, d). This transient G2/M arrest was followed by two distinct events (1) the restoration of normal cell cycle progression during the consecutive hours of the incubation with FIS, since at 30 h of the treatment, there were no significant differences in the cell cycle distribution between control and FIS-treated cells (Fig. 5a–e); (2) the appearance of the polyploid cells (DNA content greater than 4 N) but also the hypodiploid cells (DNA content lower than 2 N) at 30 h of the treatment with PTX, with or without FIS (Fig. 5a, e). The polyploid fraction significantly increased in number when FIS and PTX were administered to cells simultaneously, in comparison to the single treatment with PTX (40.52 ± 5.23 vs. 17.97 ± 8.37), what clearly indicated the potentiation of the effect of paclitaxel by fisetin (Fig. 5e). As early as 10 h of the treatment with FIS and PTX, the presence of the multinucleated interphase cells (probably tetraploid cells) that were larger than those seen in control cultures (Fig. 4h—II), accompanied by the accumulation of cells into 4 N stage (Fig. 5d) was observed. After the next round of the cell cycle, the appearance of the polyploid cells in the DNA histogram analysis (Fig. 5e) as well as the giant mononucleated or multinucleated cells under fluorescence microscope (Fig. 4l—II, III) was evident, what was consistent with the cell growth without the cell division. As mentioned above, we also noticed an increase in the mean percentage of cells with less than 2N DNA content, from 3.75 ± 1.56 in control cultures to 18.83 ± 3.41 and 22.11 ± 5.3 in PTX-only treated and co-treated populations, respectively (Fig. 5a).

In addition, to check whether the cells were able to recover to normal division cycles following the exposure to the combination of FIS and PTX, these agents were removed from the cells after 30 h of incubation and then they were maintained in a drug-free medium for the next 24 or 48 h. As shown in Fig. 5, the cells failed to regain normal proliferative potential in the time frame investigated (up to 48 h) and even most of them continued to accumulate in the polyploid state (Fig. 5e). The polyploid population at 30 h treatment (40.52 ± 5.23), increased to 52.87 ± 2.48 at 24 h post-treatment, and peaked to 71.61 ± 2.87 at 48 h post-treatment (Fig. 5e).

Since it has previously been demonstrated that the polyploid/aneuploid population produced as a result of paclitaxel exposure may undergo apoptosis in the subsequent cell cycles [33, 34], the post-treatment incubations (24 and 48 h) in a drug-free medium followed by Annexin V/PI assays were performed to study the final fate of the cells undergoing mitotic catastrophe in response to the combination of FIS and PTX. The results presented in Fig. 3b indicate that mitotic catastrophe was not followed by apoptosis at any time points examined.

Taken together, our results clearly demonstrated that FIS enhanced PTX-induced mitotic catastrophe in the A549 cells.

### Fisetin potentiated PTX-induced mitotic catastrophe through the promotion of multipolar spindle formation

**Fig. 6:**
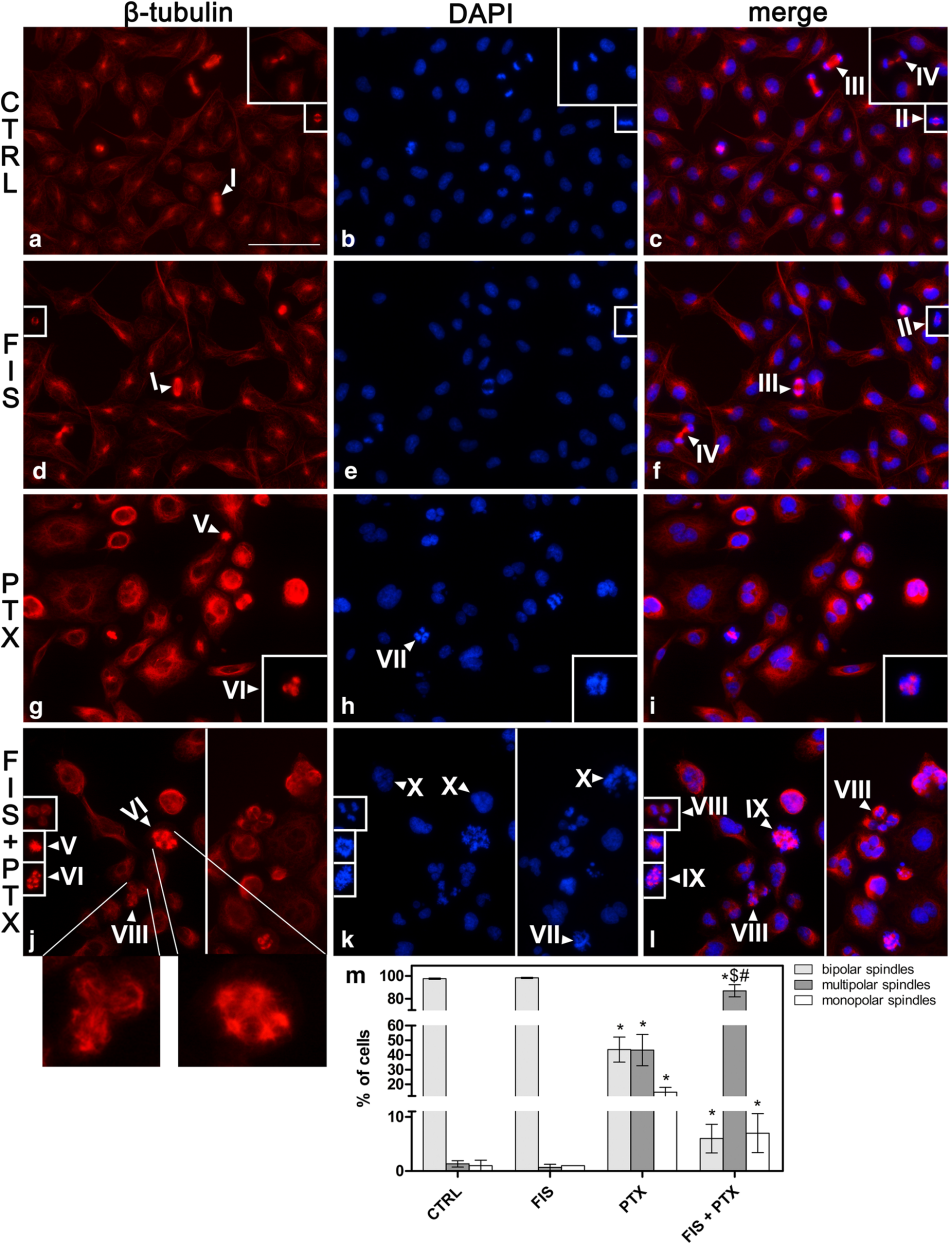
Mitosis and cytokinesis abnormalities in A549 cells after treatment with fisetin (FIS) and/or paclitaxel (PTX). The A549 cells were treated with 10 µM FIS and/or 0.1 µM PTX for 24 h or left untreated (CTRL). **a**–**l** Mitotic spindles were stained with β-tubulin antibody in *red* color and DNA was stained by DAPI in *blue* color and visualized by fluorescence microscope. *Arrowheads* indicate: normal mitotic figures in control (**a**–**c**) and FIS-treated (**d**–**f**) cells (*I*) bipolar spindles; (*II*) metaphase cells with chromosomes aligned on the metaphase plate; (*III*) anaphase cells; (*IV*) late telophase/cytokinesis; mitosis and cytokinesis defects in PTX-treated (**g**–**i**) and co-treated (**j**–**l**) cells (*V*) monopolar spindles; (*VI*) multipolar spindles; (*VII*) abnormal chromosome alignments and segregation; (*VIII*) multipolar cell division resulting from multipolar spindles; (*IX*) cleavage failure as a consequence of multipolar spindles; (*X*) multinucleated or mononucleated interphase cells as a morphological manifestation of mitotic slippage. *Bar* 50 µm. **m** The quantification of spindles abnormalities. The percentage of cells containing normal bipolar spindles (*light grey columns*), multipolar spindles (*dark grey columns*), and monopolar spindles (*white columns*) was evaluated. At least 100 cells were counted on each of the three slides. Asterisks represent statistically significant differences from control cells (p < 0.05; Mann–Whitney U test). Symbols ^$^ and ^#^ indicate statistically significant differences compared with FIS or PTX treatment alone, respectively (p < 0.05; Mann–Whitney U test)

To investigate cell-cycle-specific events that resulted in mitotic catastrophe induction under the conditions used in this study, the immunofluorescence staining of β-tubulin was performed. Since it has previously been shown that MC may be a consequence of the mitotic spindle failure, and further, since the interference with spindle microtubule dynamics is considered as a main cause of PTX-induced cytotoxicity [35], hence we asked whether such a drug effect would be enhanced by fisetin. As depicted in Fig. 6, following the 24-h exposure to paclitaxel, a significant part of the mitotic cells contained the multipolar (g—VI) or monopolar spindles (g—V) as well as the misaligned and missegregated chromosomes (h—VII), which were in clear contrast to the bipolar spindles (a, d—I), the normal metaphase plates (c, f—II), the normal anaphase progression (c and f—III) and cytokinesis (c, f—IV) seen in control and fisetin-only treated cells. The combination treatment markedly increased the incidence of the mitotic cells with the multipolar spindles (Fig. 6j—VI, m) as well as the abnormal chromosome alignments and segregation (Fig. 6k—VII, quantitative data not shown) whereas the number of cells with the monopolar spindles (Fig. 6j—V, m) was even lower when compared to the single treatment with PTX. It is important to indicate that these cells displayed either the cleavage failure (Fig. 6l—IX) or they underwent the multipolar cell division (Fig. 6j, l—VIII). The multipolar cytokinesis was associated with the asymmetric distribution of the chromatin material and resulted in several (more than two, usually three) aneuploid daughter cells (Fig. 6l—VIII). Such events were hardly ever seen in control and fisetin-only treated cultures (Fig. 6c, f). The presence of the majority of the interphase cells as the enlarged mononucleated or multinucleated cells indicated that, in the response to the combined treatment, the cells slipped from mitosis without cytokinesis (Fig. 6k—X).

Altogether, our results revealed that in co-treated cell populations, the vast majority of the mitotic cells contained the multipolar spindles that led to the cleavage failure or the aberrant cell division and produced the cells with an abnormal DNA content.

### The involvement of autophagy in the synergistic action of fisetin with paclitaxel

**Fig. 7:**
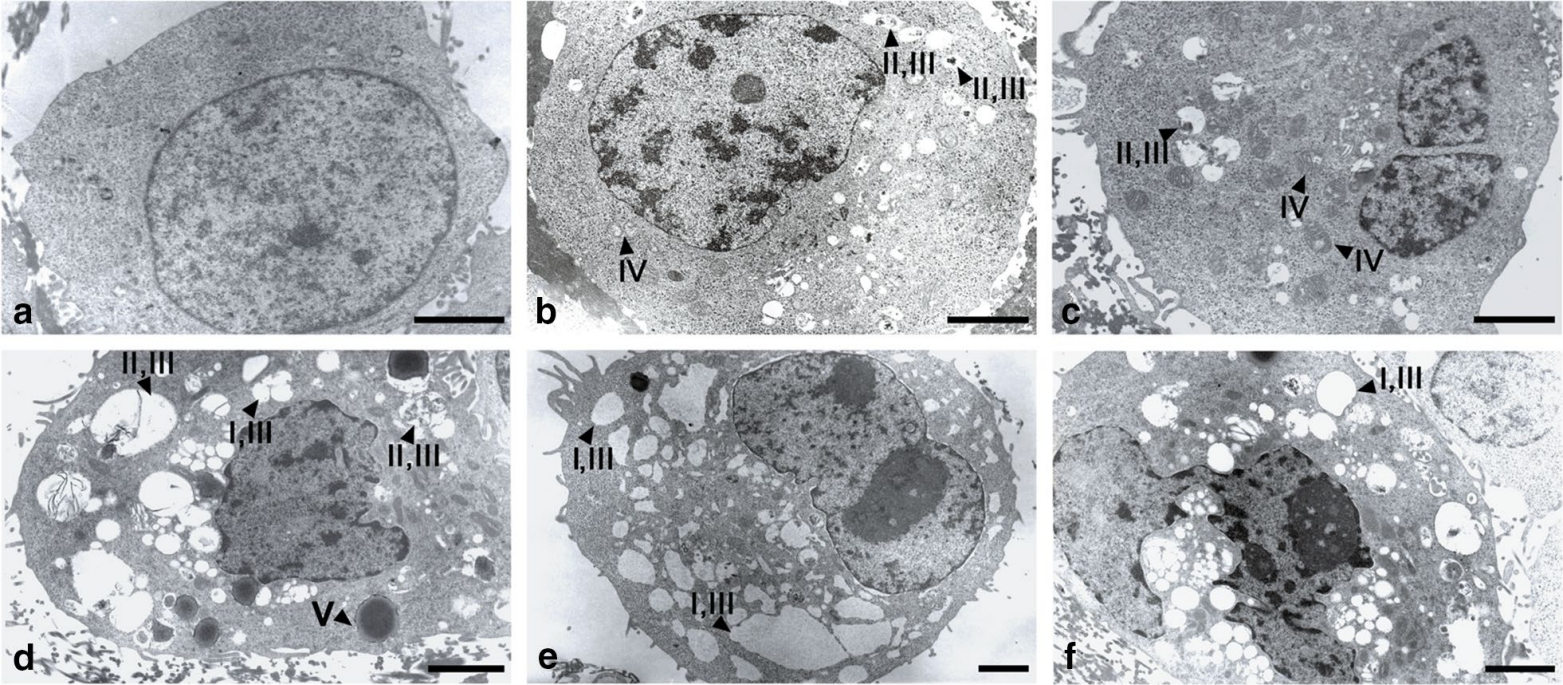
The detection of autophagy using transmission electron microscope. The A549 cells were treated with 10 µM fisetin (FIS) and/or 0.1 µM paclitaxel (PTX) for 24 h or left untreated (control, CTRL). *Arrowheads* indicate: (*I*) empty autophagic-like vacuoles; (*II*) autophagic-like vacuoles filled with the amorphous materials, the membranous inclusions or the organelles at the various stages of degradation; (*III*) single-membrane vacuoles (autolysosome-like structures); (*IV*) the swollen mitochondria with distorted or disorganized cristae; (*V*) large lysosome-like structure with electron-dense material. Representative micrographs of two independent experiments. *Bar* 2 µm

Since it has earlier been demonstrated that PTX as well as FIS may trigger autophagy in cancer cells, therefore we questioned if this process may also be involved in the synergistic action of these compounds. In the first experiment, we assessed the morphology of A549 cells at the electron microscopic level, because electron microscope is regarded as “a gold standard” in monitoring the occurrence of autophagy [36]. As shown in Fig. 7, the cells treated with either PTX or FIS exhibited the increased amount of vacuole-like structures in the cytoplasm area when compared to control cell populations. However, such an autophagic-like vacuolization appeared to be somewhat enhanced in the cells exposed to the combination of FIS and PTX (Fig. 7b–f). In other words, co-treated cells seemed to have more vacuole-like structures in comparison to control cells as well as PTX-only and FIS-only treated cells. Some of these structures were empty and huge (Fig. 7d, e—I), whereas the other were filled with the amorphous materials, the membranous inclusions and the organelles at the various stages of degradation (Fig. 7d, e, f—II). The vast majority of the observed vacuoles were limited by a single membrane (Fig. 7d, e, f—III). These changes are the hallmark features of autolysosomes, as described previously [36]. The swollen mitochondria with distorted or disorganized cristae were an additional characteristic feature of autophagy frequently seen in the cells treated with either PTX or FIS (Fig. 7b, c—IV). In these cells, similar as observed in co-treated populations, the vast majority of the autophagic vacuoles were autolysosomes (Fig. 7b, c—III). Moreover, our attention turned to the enlarged cells with extensive vacuolization (Fig. 7e), since it has previously been proposed that the cells undergoing mitotic catastrophe may die through the autophagic cell death [37]. This possibility was taken into account in our further experiments (see below).

**Fig. 8:**
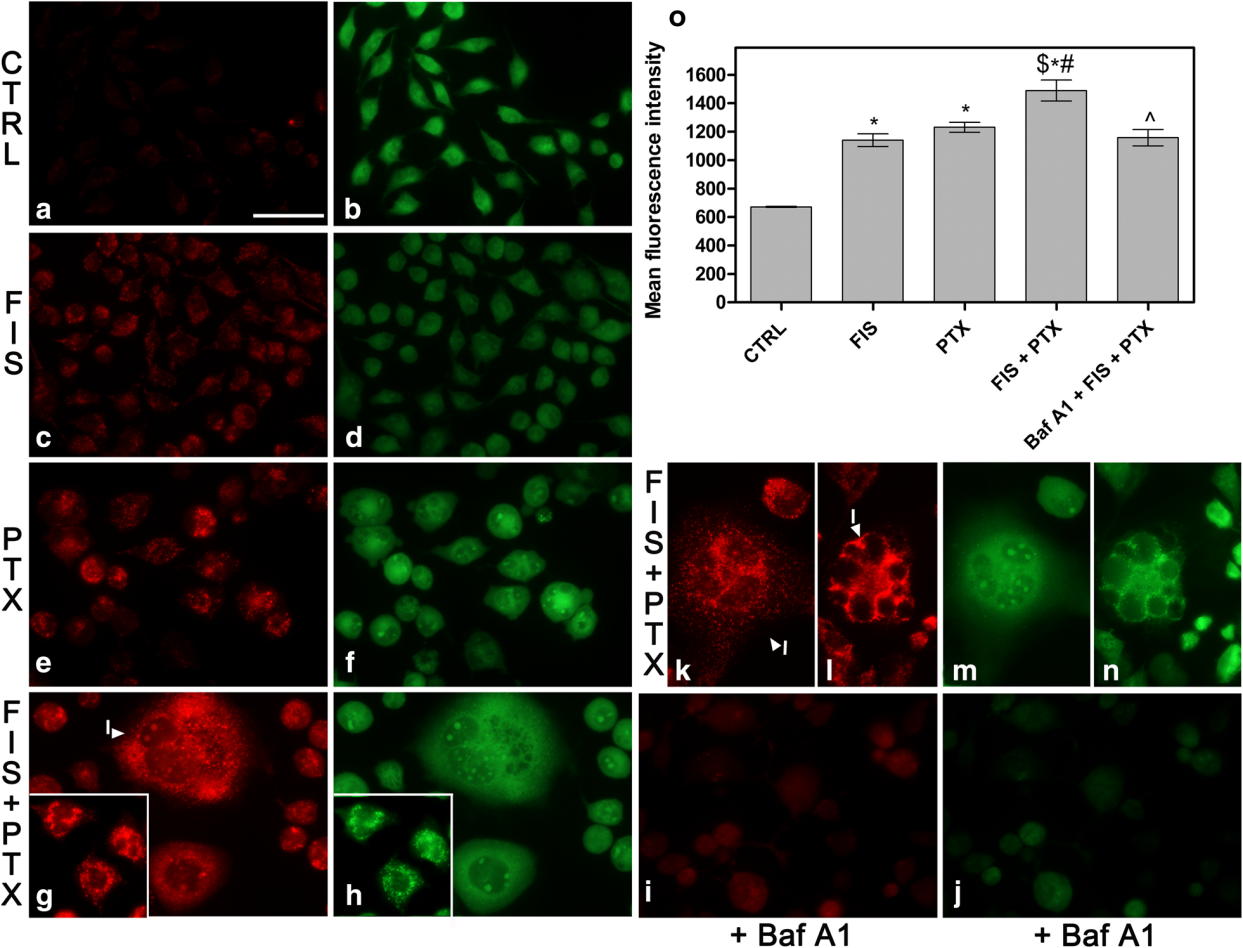
The detection of autophagy by acridine orange staining (AO). **a**–**n** Fluorescent microscope was used to visualize the acidic vesicular organelles (AVOs; *red fluorescence*) as well as the cytoplasm and nucleus (*green fluorescence*) after the vital staining of the cells with AO, as indicated in Materials and methods. **a**–**h** The A549 cells were treated with 10 µM fisetin (FIS) and/or 0.1 µM paclitaxel (PTX) for 24 h or left untreated (control, CTRL). **i**, **j** As negative control, bafilomycin A1 (Baf A1; 100 nM) was added to the cells for a period of 4 h, followed by washing with PBS and subsequent incubation with FIS and PTX for 24 h. **k**–**n** In another set of experiment, the cells were treated with the combination of 10 µM fisetin and 0.1 µM paclitaxel for 24 h, followed by post-treatment incubation in a drug-free medium for the next 24 or 48 h. Note the increased amount of AVOs after the treatment with FIS and/or PTX (**c**, **e**, **g**). *Arrowheads* indicate: (*I*) the giant multinucleated cells filled with numerous AVOs. *Bar* 50 µm. **o** The measurement of the red fluorescence of AO using image-based cytometer. Asterisks represent statistically significant differences from control cells, and symbol ^^^ indicates statistically significant differences compared to the treatment with FIS plus PTX (p < 0.05; Mann–Whitney U test). Symbols ^$^ and ^#^ indicate statistically significant differences compared with FIS or PTX treatment alone, respectively (p < 0.05; Mann–Whitney U test). Data are representative of three separate experiments and presented as mean and standard deviation

To precisely investigate if autophagy was induced under the conditions used in this study, we employed acridine orange dye (AO) which accumulates in acidic spaces, such as acidic vesicular organelles (AVOs) and emits bright red fluorescence, the intensity of which is proportional to the degree of the acidity and the volume of these structures [38]. AVOs are considered as the indicative of autophagy, therefore we assessed their presence under fluorescence microscope and then we quantitated the intensity of red fluorescence using image-based cytometer at 24 h of the treatment. As depicted in Fig. 8, the combination of FIS and PTX promoted AVOs formation to a greater extent than either agent used alone. Likewise, there was a statistically significant increase in the mean intensity of red fluorescence upon the treatment of A549 cells with FIS and/or PTX when compared to control cultures (Fig. 8o). Of note, the highest increment in the red fluorescence intensity was noted when FIS and PTX were applied to cells simultaneously, thus statistical significance occurred also between the combined treatment and the treatments with either FIS or PTX alone (Fig. 8o). An increase in the level of red fluorescent signals (reflecting an increase in AVOs formation) was significantly reduced upon the pre-treatment of cells with Baf A1 (Fig. 8i, o), the late phase autophagy inhibitor, which blocks the fusion of autophagosomes with lysosomes [39]. Furthermore, our attention was again turned toward the giant multinucleated cells, which were filled with numerous AVOs (Fig. 8g—I), therefore we also performed AO staining at 24 and 48 h post-treatment, because at these time points we observed the most intensive accumulation of polyploid cells in the response to FIS and PTX co-treatment. As we expected, the vast majority of cells were enlarged and multinucleated, and characterized by a massive formation of AVOs (Fig. 8k, l, m, n—I).

**Fig. 9:**
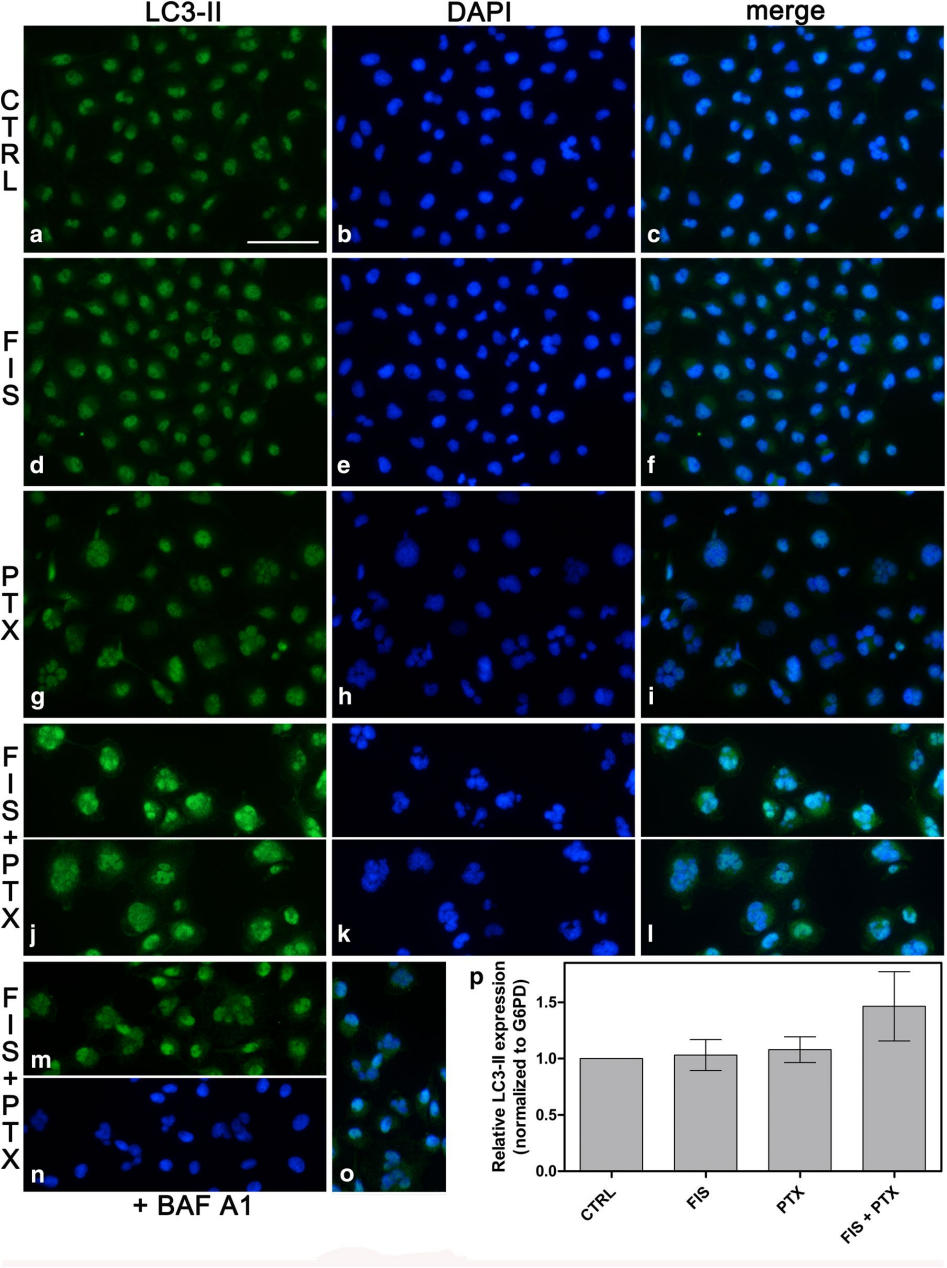
The detection of autophagy by LC3 accumulation. **a**–**o** Fluorescent microscope was used to visualize the LC3-II punctate staining pattern (*green*); the cell nuclei were counterstained with DAPI (*blue*). **a**–**l** The A549 cells were treated with 10 µM fisetin (FIS) and/or 0.1 µM paclitaxel (PTX) for 24 h or left untreated (control, CTRL). **m**–**o** As negative control, bafilomycin A1 (Baf A1; 100 nM) was added to the cells for a period of 4 h, followed by washing with PBS and subsequent incubation with FIS and PTX for 24 h. Note the increase in LC3-II puncta following FIS and PTX treatment in the absence or presence of Baf A1 (**j**, **m**). *Bar* 50 µm. **p** Real-time PCR measurement of LC3-II mRNA expression in A549 cells. The expression was normalized to glucose 6-phosphate dehydrogenase (G6PD) and presented as a fold difference relative to a calibrator sample (untreated A549 cells; designated as 1). *Error bars* represent standard deviation from duplicate qRT-PCR assays

To further corroborate the occurrence of autophagy, the effect of tested agents on LC3 immunofluorescence staining pattern was investigated. Moreover, the expression level of LC3-II was assessed using real-time PCR. LC3 is recognized as a specific marker of autophagy and it exists in two forms, cytosolic LC3-I (18 kDa) and shorter, derived from LC3-I by proteolysis and lipid modification, membrane-bound form LC3-II (16 kDa), the level of which is directly correlated with a number of autophagosomes [40]. As depicted in Fig. 9a–o, the fluorescence staining pattern of LC3 changed from weak and diffuse in control cultures to a small punctate pattern seen in fisetin and/or paclitaxel treated cells. Moreover, co-treated cells (Fig. 9j) seemed to possess more LC3-II dots than the cells exposed to FIS (Fig. 9d) or PTX alone (Fig. 9g). The accumulation of LC3-II was also observed following Baf A1 pretreatment (Fig. 9m), the effect which is consistent with the inhibition of autophagy, as described previously [41]. Next, we examined the changes in mRNA expression of LC3-II and the results are presented in Fig. 9p. After 24 h of co-treatment, the cells had increased mRNA expression of LC3-II by more than 1.5-fold. However, in the cells exposed to FIS alone or PTX alone, there was only a very slight increase in the expression level of LC3-II compared to control cultures that might be attributed to the presence mainly autolysosomes at 24 h of treatment with FIS or PTX. Indeed, it has previously been shown that when mainly late autophagic vacuoles were accumulated in the cells, LC3-II levels may be not different, or may differ only slightly from controls [42], because autophagosome-membrane-associated protein LC3-II is degraded in the aftermath of the fusion of autophagosomes with lysosomes [43]. Most likely, this was also the case of marked, but not a dramatic change, in the expression of LC3-II mRNA following the combination treatment (Fig. 9p).

Collectively, the obtained data revealed that PTX alone and FIS alone induced autophagy in A549 cells, but when used together, these agents triggered autophagy to a greater extent than either agent alone.

### Fisetin alone or paclitaxel alone induced the protective autophagy in A549 cells that switched to the autophagic cell death in the response to the combination of these agents

**Fig. 10:**
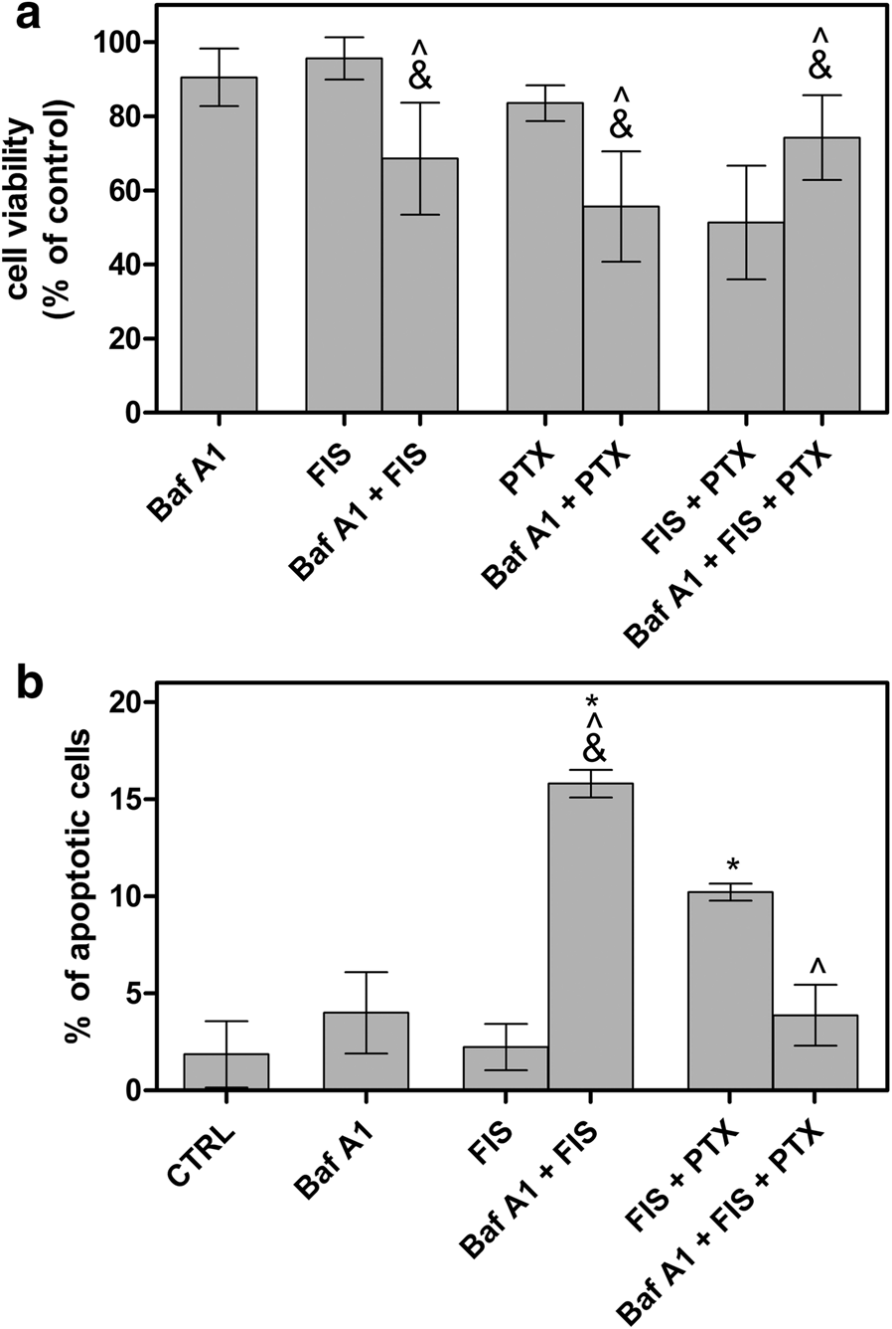
The functional significance of autophagy in A549 cells treated with fisetin (FIS) and/or paclitaxel (PTX). The A549 cells were pre-treated with bafilomycin A1 (Baf A1; 100 nM) for 4 h, followed by washing with PBS and the subsequent incubation in the absence or presence of 10 μM FIS and/or 0.1 μM PTX for 24 h. **a** Cell viability was determined by MTT colorimetric assay. Data are expressed as a percentage of the control. **b** The cytometric analysis of apoptosis using Annexin V/PI assay. The sum of the early and late apoptotic cells represented the total apoptosis. Asterisks represent statistically significant differences from control cells (p < 0.05; Mann–Whitney U test). Symbols ^&^ indicate statistically significant differences compared to the treatment with Baf A1 alone, and symbols ^^^ indicate statistically significant differences compared to the treatment with FIS alone, PTX alone, or FIS plus PTX (p < 0.05; Mann–Whitney U test). The data are representative of three independent experiments and presented as mean and standard deviation

Autophagy has recently been proposed to play a dual role in cancer therapy, either as a protective mechanism which enables cancer cell to survive under the stressful conditions or as a cancer cell death mechanism [44]. Therefore, we next aimed to determine the role of autophagy in the A549 cells treated with FIS and/or PTX. To this end, we pre-treated the cells with Baf A1 for 4 h and then again MTT assays were performed. Specifically, we were interested in whether FIS-mediated autophagy may have a cytoprotective or detrimental function, since the occurrence of autophagy in A549 has not been reported yet. As shown in Fig. 10a, the blockage of autophagy by Baf A1 significantly decreased the cell viability in the response to FIS exposure from 95.63 ± 5.70 (10 μM FIS; 24 h) to 68.56 ± 15.14 (Baf A1; 4 h, then 10 μM FIS; 24 h), suggesting that FIS-induced autophagy provided a protection and a survival advantage to A549 cells against FIS-mediated cytotoxicity. Likewise, the interference with autophagy in PTX-only treated cells led to the significant enhancement of cytotoxicity as indicated by the decline in the cell viability from 83.55 % ± 4.84 (0.1 μM; 24 h) to 55.67 % ± 14.89 (Baf A1; 4 h, then 0.1 μM PTX; 24 h) (Fig. 10a). It was an expected outcome, as paclitaxel has previously been shown to elicit the cytoprotective autophagy in the A549 cells [45]. In clear contrast, when FIS and PTX were applied simultaneously to A549 cells, Baf A1 pre-treatment resulted in the pronounced increase in the cell survival from 51.32 ± 15.39 (0.1 μM PTX/10 μM FIS; 24 h) to 74.22 ± 11.44 (Baf A1; 4 h, then 0.1 μM PTX/10 μM FIS; 24 h), indicating that autophagy was involved in the cytotoxicity of the FIS/PTX combination treatment (Fig. 10a).

It has previously been reported that the inhibition of a cytoprotective autophagy sensitizes cancer cells to an apoptotic action of chemotherapeutic agents [45], thus we questioned if autophagy blockage may increase the rate of apoptosis in FIS-treated A549 cells. As it can be seen in Fig. 10b, following Baf A1 exposure, the percentage of the Annexin V-positive cells was significantly elevated from 2.24 ± 1.92 (10 μM FIS; 24 h) to 15.81 ± 0.72 (Baf A1; 4 h, then 10 μM FIS; 24 h), indicating the protective role of autophagy against FIS-induced apoptosis. Furthermore, we were curious about whether such a negative interconnection between apoptosis and autophagy might also occur in the A549 cells exposed to the combination of FIS and PTX, in which the cytotoxic mode of autophagy was observed. As depicted in Fig. 10b, we did not observe the significant increase in the percentage of the Annexin V-positive cells following autophagy blockage in the A549 cells co-treated with FIS and PTX, when compared to the cells treated with Baf A1 alone.

Taken together, our results indicated that in A549 cells, FIS alone or PTX alone induced protective autophagy against apoptosis, however, the cytotoxic autophagy/autophagic cell death was triggered when these agents were applied simultaneously to A549 cells.

## Discussion

In the present paper, we reported the first experimental evidence for the existence of synergism between fisetin and paclitaxel in the in vitro model of NSCLC. This synergism was quantified by the combination index method of Chou and Talalay [19], which is based on the multiple drug effect equation derived from the median-effect principle of the mass-action law [21]. Such preclinical drug combination studies in vitro and/or in animals are necessary to obtain the basis and rationale for the drug combination clinical trials, since from scientific, practical and ethical reasons it is impossible to determine synergism in humans [46]. However, to avoid a lack of reproducibility of the in vitro tests in the clinical trials, as it is often seen in the clinical settings, an important issue for the in vitro testing of agents is the use of clinically relevant concentrations, on condition that the pharmacokinetic data are available [47]. With this in mind, our intention was to use the in vivo attainable concentrations of fisetin (≤20 µM) [3, 24] to find its synergistic combinations with clinically achievable doses of cytostatic drugs, such as paclitaxel, mitoxantrone, methotrexate or arsenic trioxide. According to the literature reports, the doses of PTX below or equal to 1 µM [25, 26], MIT below or equal to 1 µM [48], MTX below or equal to 1 µM [49], and ATO below or equal to 2 µM [50] are achievable in vivo and clinically relevant. Therefore, although our studies were carried out in vitro, all drugs were tested at the plasma concentrations attainable in vivo, providing a high hope for their easy reproducibility into the clinical settings.

In the present studies, the CI analysis demonstrated that the combination effect of FIS and PTX was synergistic in the A549 non-small lung cancer cells, in all concentrations tested and over a wide range of effect levels (f_a_). The highest degree of synergism was found when 10 μM FIS was combined with 0.1 μM PTX (CI = 0.15), therefore we selected these doses for further drug combination studies on mechanistic aspects. In accordance with the previous report of Liao et al., we have shown that 10 μM FIS itself was not toxic to A549 [51]. However, the contrary results have been obtained by Khan et al., who have revealed that the treatment with 10 µM fisetin for 24 h decreased the viability of A549 cells by 25 %, as measured using MTT assay [3]. Although the reasons for such a significant discrepancy are unclear, the differences in the experimental methodology, e.g. in the MTT assay and the cell treatment (at different degrees of confluence) may be, at least partially, responsible. In the case of paclitaxel, we noticed that 0.1 μM concentration of this drug failed to substantially decrease the viability of A549 cells, allowing ~83 % of cells to remain viable, the results of which are in agreement with earlier reports from this cell line (for example, the IC_50_ values of PTX in A549 cells was determined to be 5 μM [27], 22.5 μM [28] or 8.20 μM [29].

Here, we also found that the combination effect of FIS and PTX was cell line-specific. Synergy was demonstrated in the LoVo and H1299 cells but not in the MCF-7 cells, where the strong antagonism was observed. In the LoVo and H1299 cells, the synergism was seen in a narrow range of effect levels, which, in the case of the latter cells may be considered as irrelevant from a clinical perspective, because they represent only a minor growth inhibition (IC_38_). The genetic differences among the tested cell lines that contributed to the diversity in the obtained interaction patterns are not known for the moment. A simple explanation that a diverse sensitivity might arise from different p53 status of the used cell lines, has rather been dismissed, because A549 and MCF-7 cell lines, both having functional p53, exhibited an opposite interaction pattern. However, we cannot firmly rule out this account since A549 (wild-type p53) and H1299 (p53-deficient) cells, representing two histopathological subtypes of lung cancer, also exhibited a distinct interaction pattern, and simultaneously it is possible that in the MCF-7 cells, the antagonistic interactions between FIS and PTX might be determined by other factors. Future studies will have to address this issue since such information may determine the potential clinical utility of FIS/PTX in the treatment of cancer. A cell-type dependent response to drugs implies the necessity to explore genes/molecular pathways that determine the chemosensitivity of tested cell lines. From a clinical perspective, it means that careful consideration should be paid to each patient’s individual characteristics to choose the most beneficial drug combination.

To date, only several studies have evaluated the fisetin’s ability to potentiate the anticancer activity of classical chemotherapeutic agents. It has been shown that fisetin enhances the cytotoxicity of cisplatin, TNF and doxorubicin in the H1299 non-small cell lung cancer cells [52]. Importantly, fisetin has also been reported to act synergistically with cisplatin in vivo, in the NT2/D1 mouse xenograft model [37]. In the cited study, the extent of tumor regression achieved with the co-treatment of fisetin (1 mg/kg/day) and cisplatin (1.5 mg/kg/day) was significantly more than the monotherapeutic treatments, whereas no toxicity was detected. Furthermore, using Lewis lung carcinoma-bearing mice as an experimental model, Touil et al. have shown that when fisetin was combined with cyclophosphamide, a marked improvement in the anticancer and antiangiogenic activity was observed (92 % tumor growth inhibition and a significant reduction in the microvessel density), with a low systemic toxicity [53]. However, to our knowledge, neither methotrexate nor mitoxantrone and arsenic trioxide have not been previously tested in the combination with fisetin. In the present study, we demonstrated that, while the combinations of FIS with MTX and especially with MIT do not deserve further attention (at least in the case of A549 cells), its strong synergistic action with ATO on the contrary does.

Here, we also provided some insights into the mechanism of the synergistic action of FIS and PTX in the A549 non-small cell lung cancer cells. Firstly, we proposed that mitotic catastrophe, rather than apoptosis, was one of the possible mechanism of the synergistic cytotoxicity between fisetin and paclitaxel. While some of the characteristic features of apoptosis were noticed, such as phosphatidylserine externalization, the increase in caspase-3 mRNA level, the nuclear shrinkage and fragmentation, the level of these changes was relatively low and could not account for the extent of cell death triggered by the combination of FIS and PTX. Instead, major cellular events associated with mitotic catastrophe, including G2/M arrest followed by the mitotic slippage and polyploidy/aneuploidy, as well as the chromosome misalignment and missegregation and the appearance of the enlarged mononucleated and multinucleated cells were predominantly observed in the cell populations treated with PTX alone and, to a greater extent, in the FIS/PTX co-treated cells. The polyploid/aneuploid cells were most likely the result of the multipolar spindle formation that led to either the cleavage failure or the multipolar cell division. The asymmetric cytokinesis following the multipolar mitosis resulted in the generation of three or more daughter cells with an abnormal DNA content, including the hypodiploid cells (DNA content lower than 2 N) that appeared in the sub-G1 peak of the DNA histogram. Indeed, it has previously been shown that the cells undergoing multipolar and asymmetric divisions may then have an average of 1.33N DNA instead of 2N DNA [54]. In turn, the cytokinesis failure was manifested morphologically as the enlarged mononucleated or multinucleated cells that probably were represented by the G2/M peak (as a consequence of one round of aberrant mitosis) or the polyploid fraction (that arose from another round of aberrant mitosis) on the DNA histograms. In fact, the G2/M peak, consists of both the mitotic cells (mitotic arrest) and the postmitotic tetraploid cells, which escape the mitotic block without cytokinesis [55]. Based on the fact that multinucleation is the hallmark of the mitotic slippage [55, 56], we can presume that an increased accumulation of G2/M phase cells at 10 h after starting of the treatment with FIS plus PTX, was a result of the generation of postmitotic tetraploid cells (rather than mitotically arrested cells), since at this time point, we simultaneously observed a massive accumulation of the enlarged multinucleated cells. To determine the percentage of the mitotic cells, a dual-color flow cytometry analysis using for example PI and mitotic phosphoprotein monoclonal-2 (MPM-2) or phospho-histone H3 antibody should be performed in the future studies. The above-mentioned events, all of which are related to MC, have previously been observed in various PTX-treated cancer cell lines [57, 58], including A549 cell line [59], as well as in the response to other microtubule-stabilizing drugs [60]. Although, the final outcome of mitotic catastrophe is the cell death, the time in which cells die, the ultimate cause of the cell death as well the type of a cell death pathway they follow to die, may vary in depending on the genetic background of cells and the type of drug as well as the dosage and the duration of treatment [61]. At this point, it should be emphasized that in an international consensus mitotic catastrophe is defined as an oncosuppressive mechanism occurring during or after a faulty mitosis leading to the cell death, which takes place via apoptosis or necrosis, rather than cell death executioner pathway itself [62, 63]. It has been shown that paclitaxel induces the activation of spindle assembly checkpoint through the suppression of microtubule dynamics, leading to a prolonged mitotic arrest before anaphase, followed by apoptosis [64] or eventually the mitotic exit due to either the checkpoint adaptation [65] or perturbation [57]. The resultant multinucleated G1-tetraploid cells subsequently arrest in postmitotic G1, as a result of the activation of a p53-dependent G1 checkpoint, whereas the arrest at the metaphase–anaphase transition and the mitotic slippage are most likely not mediated by p53 [66, 67]. Those cells either succumb directly to apoptosis (mitotic catastrophe followed by apoptosis) or continue another round of the cell cycle as a consequence of the G1 checkpoint failure. The latter event, leads to further polyploidization and aneuploidization and eventually to the cell death [57, 59]. Importantly, herein we demonstrated that PTX-induced cellular events associated with mitotic catastrophe was significantly potentiated by fisetin. Bearing in mind that A549 cells have a wild type p53 gene, one can ask why the post-slippage A549 cells did not arrest in the G1 postmitotic checkpoint, and instead entered another round of the cell cycle to form the polyploid progeny and how to account for the reported potentiation of PTX-induced mitotic catastrophe by fisetin. One plausible explanation comes from the observation that fisetin may act as a strong inhibitor of the spindle checkpoint that induces a rapid escape from microtubule drug-induced mitotic arrest [68]. In the cited studies, fisetin at concentration of 30 µM caused a forced mitotic exit from the mitotic block triggered by nocodazole, taxol and monastrol in various human cancer cell lines, including A549 cells. Furthermore, there is evidence that not only p53 but also a prolonged spindle checkpoint-mediated mitotic arrest is required for the postmitotic G1 checkpoint function [67]. The duration of mitotic arrest has been shown to be critical for the stabilization and activation of p53 [55, 67]. Indeed, Vogel et al. have demonstrated that in spindle checkpoint compromised cells, mitotic arrest is shortened, resulting in subsequent endoreduplication, whereas extending the time of mitotic block in these cells inhibited endoreduplication [67]. Based on the above findings, we can speculate that the potentiation of PTX-induced mitotic catastrophe by fisetin may be associated with the perturbation of the spindle assembly checkpoint.

There are also several studies that reported on other than necrosis, non-apoptotic mechanisms leading to the cell death following mitotic catastrophe. In accordance with our results, Kuwahara et al. have suggested that the giant multinucleated cells may die through the autophagic cell death [69]. In our studies, mitotic catastrophe was not followed by apoptosis at any time points examined. Instead, we observed that the cells with the mitotic catastrophe-like phenotype were filled abundantly with autolysosomes, what allows us to assume that these cells could be eliminated through autophagy.

The role of autophagy in cancer therapy raised a paradox wherein, on one hand, it can represent a protective mechanism that sustains tumor cell growth and survival in the face of the toxicity of the cytostatic drugs or radiation, but on the other hand it may constitute an alternative form of the programmed cell death, named the autophagic type II cell death [70]. In the first scenario, autophagy contributes to the treatment failure, thus its inhibition can re-sensitize previously resistant cancer cells to the cytotoxic action of chemotherapy or radiotherapy, concurring to beneficial treatment outcome [54]. In the latter case, autophagy may be therapeutically desired, as it mediates the cytotoxic effect of anticancer drugs, leading to tumor cell demise [71]. The dual role of autophagy, either as pro-survival or pro-death mechanism, creates the need to carefully examine the functional status of autophagy before the administration of autophagy-induced therapy. In other words, from a therapeutic perspective, it is extremely important to determine whether the increase in the autophagy level is a sign of responsiveness or resistance to the treatment [72]. Hence, in the current studies, having established that FIS and/or PTX trigger autophagy in A549 cells, we then asked whether FIS and/or PTX-mediated autophagy may have a cytoprotective or detrimental function. Recent studies have shown that the impact of PTX on autophagy may be cell type-specific [73], and several reports have revealed that PTX-induced autophagy in the A549 cells represents a self-defense mechanism that protects these cells against PTX-mediated apoptosis [45]. Our observations were also consistent with the premise that autophagy induced by paclitaxel in the A549 cells is cytoprotective. In turn, to our knowledge, there is only one published study that examined the functional significance of FIS-triggered autophagy. That study has revealed that fisetin promotes the autophagic cell death in the PC3 prostate cancer cells [74]. On the contrary, we demonstrated that FIS-elicited autophagy provides a survival advantage to the A549 cells and protects them against apoptosis induced by this flavonoid. It should be emphasized that fisetin produced the protective autophagic response in the A549 cells at in vivo achievable concentration (10 μM), whereas the autophagic cell death in the PC3 cells was induced by much higher doses of fisetin (40–120 μM) [74]. These results seem to support a more and more common opinion that a potential utility of the dietary polyphenols in anticancer therapy lies in the synergistic combinations rather than in monotherapy. Interestingly, the conversion of the autophagic function from the cytoprotective form with FIS alone or PTX alone to the cytotoxic form upon the exposure of the cells to the combination of these compounds was found to occur in our experimental conditions. Indeed, when FIS and PTX were applied simultaneously to the A549 cells, Baf A1 pre-treatment resulted in the restoration of the cell viability to the level similar to what was observed with PTX treatment alone. Among the criteria adopted to define the cell death by autophagy, the demonstration that a pharmacologic or genetic suppression of autophagy prevents cell death, is believed to be the critical one [75, 76]. This type of “autophagic switch” has been first demonstrated by Wilson et al., who have shown that autophagy can actually have dual functions in the same experimental system (breast tumor cells), acting both as a cytoprotective mechanism for radiation alone and a cytotoxic mechanism when radiation is accompanied by vitamin D or 1.25 dihydroxy vitamin D3 [77]. The cited authors have implicated the autophagic cell death in the mechanism underlying the radiosensitization by 1.25 dihydroxy vitamin D3. In another study, Gewirtz’s group have utilized vitamin D and its analogue in an effort to improve the effectiveness of radiation therapy in non-small cell lung cancer [78]. As in the breast tumor cell studies, the switch between a cytoprotective and cytostatic autophagy appeared to mediated the sensitization to radiation. However, the specific signaling pathways mediating this dual role of radiation-induced autophagy have not been established so far [77, 78]. Despite the fact that we also did not investigate the molecular mechanism governing the “autophagic switch” in our experimental conditions, we presume that the significant intensity of the autophagy level in the response to the combination treatment could be, at least in part, a cause for such conversion. This assumption was based on the previous suggestions that a basal enhanced level of autophagy in tumor cells contributes to therapy resistance but a prolonged and excess induction may result in cell death by cellular self-degradation [79, 80].

The concept of “molecular switches” has also been used in the literature to describe the crosstalk between apoptosis and autophagy [80]. Although the functional relationship between these two processes has not been fully clarified, it has been suggested that autophagy and apoptosis may occur independently of each other, cooperate or antagonize each other [81]. As mentioned above, in this paper we revealed that autophagy induced by FIS alone protects the A549 cells against FIS-promoted apoptosis. In this case, the cytoprotective function of autophagy was mediated through a negative modulation of apoptosis. However, there are also reports showing that the inhibition of a cytotoxic autophagy switched cell death to apoptosis in cancer cells [82]. Therefore, we were curious about whether such a cross-regulation between autophagy and apoptosis might also occur in the A549 cells exposed to the combination of FIS and PTX. We did not observe a significant increase in the percentage of the apoptotic cells following autophagy blockage in the A549 cells co-treated with FIS and PTX, thus we suppose that in the case of the combined treatment, the autophagic and apoptotic cell death are not related to each other.

## Conclusions

In summary, here we demonstrate that fisetin synergizes with paclitaxel in A549 non-small lung cancer cell line at concentrations achievable in vivo. We also found that the possible mechanisms of this synergism involve (1) the induction of mitotic catastrophe probably through the promotion of multipolar spindle formation (2) the elimination of the cells with mitotic catastrophe through autophagy (3) a substantial increase in the level of autophagy, which presumably underlies the switch from the cytoprotective autophagy (elicited by FIS alone or PTX alone) to the autophagic cell death. These findings seem to be relevant in the light of the current understanding that the manipulation of autophagy in favor of the inhibition of its cytoprotective effects and the induction of the autophagic cell death might be a promising approach for anticancer therapy. Furthermore, since the resistance to apoptosis is the primary obstacle in the cancer treatment, the development of novel agents that elicit the non-apoptotic cell death pathways is now recognized as a prospective direction towards overcoming this limitation [17, 54]. Therefore, our results deserve further preclinical studies to explain in detail the mechanisms involved in the synergistic action of fisetin and paclitaxel. Simultaneously, the potential clinical utility of the obtained results is highly dependent on the evaluation of the efficacy and toxicity of the combination of fisetin and paclitaxel in an animal model in vivo.

## Additional file

10.1186/s12935-016-0288-3 The combined effect of fisetin with mitoxantrone (MIT), or methotrexate (MTX), or arsenic trioxide (ATO). (a,b) Logarithmic combination index plot (Fa-log(CI) plot) for FIS and MIT or MTX, respectively. CI values (logarithmic) are plotted as a function of the fractional inhibition (fa) of cell viability/growth by computer simulation (CompuSyn software) from 0.1 to 0.95. In the Fa-log(CI) plot, the synergism is indicated by a negative value (log(CI) < 0), antagonism is indicated by a positive value (log(CI) > 0), and additive effect (denoted by a dashed line) is indicated by 0 (log(CI) = 0). (c) Combination index plot (Fa-CI plot) for FIS and ATO co-treatment. CI values are plotted as a function of the fractional inhibition (fa) of cell viability/growth by computer simulation (CompuSyn software) from 0.1 to 0.95. CI < 1 designates synergism, CI = 1 indicates additivity (denoted by a dashed line), and CI > 1 represents antagonism. In all cases triangles represent CI values derived from the actual experimental points.

## Abbreviations

AO: acridine orange
AVOs: acidic vesicular organelles
Baf A1: bafilomycin A1
BSA: bovine serum albumin
CI: combination index
DAPI: 4′,6-diamino-2-phenylindole
D_*m*_: the dose that produces 50 % effect, such as IC_50_
DMEM: Dulbecco’s Modified Eagle Medium
f_a_: fraction affected (the fraction of cells inhibited after the drug exposure, e.g. 0.5 when cell growth is inhibited by 50 %)
FIS: fisetin
G6PD: glucose 6-phosphate dehydrogenase
HBSS: Hank’s balanced salt solution
IC_50_: half maximal inhibitory concentration
*m*: the slope of the median-effect plot, which indicates the shape of the dose–effect curve
MC: mitotic catastrophe
MEM: Minimum Essential Medium
MPM-2: mitotic phosphoprotein monoclonal-2
MTSB: microtubule stabilizing buffer
MTT: thiazolyl blue tetrazolium bromide
NSCLC: non-small cell lung cancer
PBS: phosphate-buffered saline
PI: propidium iodide
PSA: prostate-specific antigen
PTX: paclitaxel
qRT-PCR: real-time quantitative reverse transcription PCR
*r*: linear correlation coefficient, which describes the conformity of the data to the median-effect plot of the mass-action law
RPMI 1640: Roswell Park Memorial 1640 medium
RT: room temperature
SCLC: small-cell lung cancer
SD: standard deviation
TBS: tris-buffered saline
TEM: transmission electron microscope
TNF: tumor necrosis factor
