# Supplementary figures and images for: Paclitaxel and the dietary flavonoid fisetin: a synergistic combination that induces mitotic catastrophe and autophagic cell death in A549 non-small cell lung cancer cells
Source: Cancer Cell Int. 2016 Feb 16;16:10. doi: 10.1186/s12935-016-0288-3 (PMC4754822; doi:10.1186/s12935-016-0288-3)

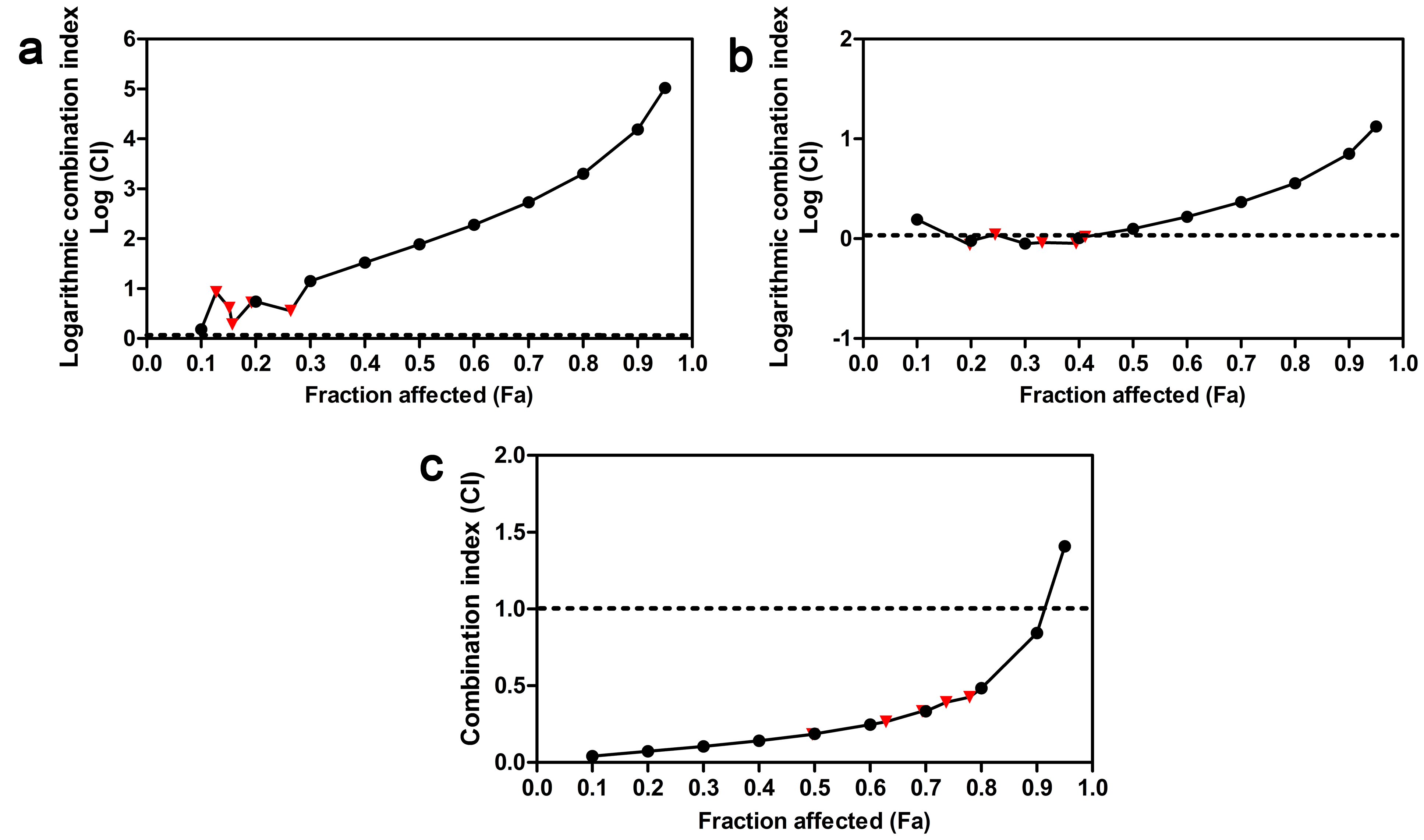

Supplement: Supplementary file 1 — 10.1186/s12935-016-0288-3 The combined effect of fisetin with mitoxantrone (MIT), or methotrexate (MTX), or arsenic trioxide (ATO). (a,b) Logarithmic combination index plot (Fa-log(CI) plot) for FIS and MIT or MTX, respectively. CI values (logarithmic) are plotted as a function of the fractional inhibition (fa) of cell viability/growth by computer simulation (CompuSyn software) from 0.1 to 0.95. In the Fa-log(CI) plot, the synergism is indicated by a negative value (log(CI) < 0), antagonism is indicated by a positive value (log(CI) > 0), and additive effect (denoted by a dashed line) is indicated by 0 (log(CI) = 0). (c) Combination index plot (Fa-CI plot) for FIS and ATO co-treatment. CI values are plotted as a function of the fractional inhibition (fa) of cell viability/growth by computer simulation (CompuSyn software) from 0.1 to 0.95. CI < 1 designates synergism, CI = 1 indicates additivity (denoted by a dashed line), and CI > 1 represents antagonism. In all cases triangles represent CI values derived from the actual experimental points. [file 12935_2016_288_MOESM1_ESM.tif]
